# Comparative evaluation of cardiovascular risks among nine FDA-approved VEGFR-TKIs in patients with solid tumors: a Bayesian network analysis of randomized controlled trials

**DOI:** 10.1007/s00432-021-03521-w

**Published:** 2021-03-16

**Authors:** Wanting Hou, Mingfu Ding, Xiaohua Li, Xiaohan Zhou, Qing Zhu, Armando Varela-Ramirez, Cheng Yi

**Affiliations:** 1 Department of Medical Oncology, Cancer Center, West China Hospital, Sichuan University, Sichuan, China; 2 Department of Dermatovenereology, West China Hospital, Sichuan University, Sichuan, China; 3 Department of Biological Sciences, The Border Biomedical Research Center (BBRC), The University of Texas At El Paso, El Paso, TX 79968 USA; 4 Department of Rehailitation, West China Hospital, Sichuan University, Chengdu, China

**Keywords:** VEGFR-TKI, Solid tumor treatment, Cardiovascular risk, Bayesian meta-analysis

## Abstract

**Purpose:**

The present meta-analysis study was performed to identify the potential cardiotoxicity risks when using Vascular Endothelial Growth Factor Receptor Tyrosine kinase inhibitors (VEGFR-TKIs) as anticancer drugs in patients with solid tumors.

**Methods:**

Pubmed, Embase, the Cochrane Central Register of Controlled Trials, and ClinicalTrials.gov databases were searched for the randomized controlled trials. We have included 45 randomized controlled trials (RCTs) associated with nine VEGFR-TKIs Food and Drug Administration (FDA)-approved drugs used to treat patients with solid tumors. To evaluate the trials’ risk of bias, Cochrane Risk of Bias Tool was assessed. A direct comparison was assessed by RevMan5.3 software, calculating the odds ratio (OR) and 95% confidence interval (CI). Heterogeneity was tested by the *I*^2^ statistic and Chi-square test for *P* value. Bayesian network meta-analysis was performed using Stata 15.0 and GeMTC 0.14.3 software, calculated OR along with corresponding 95% credible interval (CrI). The model’s convergence was evaluated by the potential scale reduced factor (PSRF). Consistency between direct and indirect comparisons was assessed by the “node-splitting” method.

**Results:**

In this network meta-analysis, a total of 20,027 patients from 45 randomized controlled trials and associated with nine FDA-approved VEGFR-TKIs (axitinib, cabozantinib, lenvatinib, nintedanib, pazopanib, regorafenib, sorafenib, sunitinib, vandetanib), were enrolled. Findings indicated that lenvatinib had the most significant probability of provoking all grades cardiovascular incident and hypertension, followed by vandetanib, cabozantinib, axitinib, pazopanib, sorafenib, sunitinib, regorafenib and nintedanib. The nine agent’s severe cardiovascular and severe hypertension risk was probably similar. The ranking probability of cardiac toxicity shows that vandetanib ranked most likely to have the highest risk for cardiotoxicity among all the VEGFR-TKIs reviewed, followed by pazopanib, axitinib, sorafenib, sunitinib. In contrast, regorafenib and nintedanib did not exhibit an increased risk of cardiac damage.

**Conclusions:**

The association between the nine VEGFR-TKIs with potential cardiotoxicity occurrence was reviewed. Both the regorafenib and nintedanib did not display detectable signs of cardiotoxic damage. In contrast, lenvatinib and vandetanib are ranked to have the most severe cardiotoxicity side impacts. These results may provide information for clinical practice guidelines, implementing strategies in selecting the adequate VEGFR-TKIs, and understanding the cardiovascular toxicity inflicted by the VEGFR-TKIs.

**PROSPERO identifier:**

CRD 42,020,167,307.

**Supplementary Information:**

The online version contains supplementary material available at 10.1007/s00432-021-03521-w.

## Introduction

Tumor blood vessels provide nutrients and oxygen for solid tumor growth and metastasis (Kerbel 2000). Anti-angiogenesis drugs have shown efficacy in many solid tumor patients’ treatments. Vascular endothelial growth factor (VEGF) plays an essential role in tumor angiogenesis. Blocking this signal pathway can effectively inhibit tumor angiogenesis (Verheul and Pinedo 2000). Vascular Endothelial Growth Factor Receptor Tyrosine kinase inhibitors (VEGFR-TKIs) were small molecule drugs that targeted VEGFR. Some VEGFR-TKIs have shown clinical efficacy in treating many types of solid tumors (Abou-Alfa et al. 2018; Gounder et al. 2018; Llovet et al. 2008). Treatment with VEGFR-TKIs combined with another anti-tumor therapy (chemotherapy, radiotherapy, etc.) also was used in solid tumor treatment in recent years (Park et al. 2019; Samalin et al. 2019; Wilky et al. 2019). There are nine VEGFR-TKIs had been approved by the Food and Drug Administration (FDA-US), including regorafenib, vandetanib, cabozantinib, lenvatinib, axitinib, sunitinib, sorafenib, nintedanib, and pazopanib. Adverse frequencies have been frequently reported after using these VEGFR-TKIs; cardiovascular toxicities are the inevitable VEGFR-TKIs-provoked side effect.

A functional VEGF signal pathway is essential in healthy vasculature growth and development (Ribatti 2019). VEGFR-TKIs’ cardiovascular toxicities include both vascular and cardiac side effects. The most common adverse effect observed in patients receiving VEGFR-TKIs are hypertension, cardiac ischemia, QT prolongation, arterial thromboembolic illnesses, and venous thrombosis. Cardiovascular severe adverse disorders not only cause patient’s therapy discontinuation but also threaten patients’ lives. Thus, it is of great importance to clarify the cardiovascular toxicity of VEGFR-TKI agents.

The previous meta-analysis had discussed the VEGFR-TKIs’ cardiovascular risk from different aspects, but some of them reported conflicting results, and some reports’ samples were small. There are already four meta-analyses which focus on VEGFR-TKIs cardiovascular toxicity. In the first study, performed by Abdel-Qadir et al. (2017), they found that the VEGF inhibitors were associated with an increased risk of arterial thromboembolism OR = 1.52 (OR odd ratios), 95% confidence interval (CI) 1.17–1.98. The second, conducted by Li et al. (2018), suggests that VEGFR-TKIs significantly increases the risk of all-grade and high-grade hypertension, all-grade bleeding, and all-grade cardiac dysfunction. But no significant increased risk of all-grade and high-grade thromboembolism, high-grade bleeding, and high-grade cardiac dysfunction associated with these agents. The third study, performed by Totzeck et al. (2018), found that tyrosine kinase inhibitor treatment was associated with a higher cardiac ischemia relative risk (RR 1.69, 95% CI 1.12–2.57), the most top risk was observed when using sorafenib for patients with renal cancer. Left ventricular systolic dysfunction was increased after tyrosine kinase inhibitor therapy (RR 2.53, 95% CI 1.79–3.57), the highest hazard was reported in sunitinib for hepatocellular cancer. QT corrected interval prolongation (RR 6.25, 95% CI 3.44–11.38), and arterial hypertension (RR 3.78, 95% CI 3.15–4.54) were also reported. A similar RR of arterial adverse effects, cerebral ischemia, adverse venous conditions, and pulmonary embolism were found across groups. The fourth study, conducted by Furuya et al. (2020)**.** By re-examining the meta-analysis performed by Totzeck et al. (2018), they thought that the VEGFR-TKIs were associated with a small increase hazard of patients developing hypertension, arterial thrombotic damage, thrombocytopenia, and bleeding.

Additionally, there are three meta-analyses of VEGFR-TKIs-related arterial thromboembolic or venous thrombosis risk incidence. The first, conducted by Qi et al. (2013), compared the risk of VEGFR-TKIs’ (pazopanib, sunitinib, sorafenib, and vandetanib) with venous thromboembolic events (VTEs), and found that use of VEGFR-TKIs does not significantly increase the risk of VTEs. The second was performed by Sonpavde et al. (2013). The results show the RR of all grade and high-grade VTEs for the TKI vs. no TKI arms were 1.10 (95% CI 0.73–1.66) and 0.85 (95% CI 0.58–1.25), respectively. The third report compared the risk of VTEs and arterial thromboembolic occurrences (ATEs) associated with VEGFR-TKIs (sorafenib, sunitinib, pazopanib, vandetanib, axitinib, cediranib, regorafenib, linifanib, motesanib, cabozantinib, dovitinib, nintedanib) (Liu et al. 2017a). In this meta-analysis, VEGFR-TKIs did not significantly increase the hazard of developing all-grade and high-grade VTEs. The use of VEGFR-TKIs substantially increases the risk of developing all-grade ATEs, and a tendency to increase the risk of high-grade ATEs (RR 1.49, 95% CI 0.99–2.24). In the meta-analysis of VEGFR-TKIs’ hypertension risk in cancer patients (Liu et al. 2016). VEGFR-TKIs remarkably enhanced the venture of developing high-grade (RR 4.60, 95% CI 3.92–5.40) and all-grade (RR 3.85, 95% CI 3.37–4.40) hypertensive effects. In a meta-analysis of QTc interval prolongation with VEGFR TKIs (sunitinib, sorafenib, pazopanib, axitinib, vandetanib, cabozantinib, ponatinib, and regorafenib) (Ghatalia et al. 2015a). The RR for all-grade and high-grade QTc prolongation for the TKI vs. no TKI arms were 8.66 (95% CI 4.92–15.2) and 2.69 (95% CI 1.33–5.44), respectively. Further subgroup analysis showed that both sunitinib and vandetanib were associated with a statistically significant risk of QTc prolongation; higher doses of vandetanib was associated with a more significant hazard. The meta-analysis of congestive heart failure (CHF) with VEGFR-TKIs usage was also detected. The RR of all grade and high-grade CHF for the TKI arm was 2.69 (95% CI 1.86–3.87) (Ghatalia et al. 2015b).

The network meta-analysis (NMA) is the extension of pairwise meta-analysis. The NMA provides a method for comparisons between all available interventions, facilitates indirect comparisons of multiple interventions, which have not been studied in a head-to-head fashion, and the NMA produces a relative ranking of all treatments (Mills et al. 2013). There are two methods for NMA: The Frequentist approach and Bayesian approach. The advantage of the Bayesian approach is that comparison treatments can be ranked for overall effectiveness based on a *priori assumptions* (Salanti et al. 2011). Herein, we aimed to compare the cardiovascular risk of VEGFR-TKIs for patients with solid tumors based on the Bayesian network meta-analysis methodology.

## Methods

This meta-analysis is performed according to the extension of the PRISMA (Preferred Reporting Items for Systematic Reviews and Meta-analyses) statement for reporting of systematic reviews incorporating network meta-analyses statements (Hutton et al. 2015). The study was registered with a prospective international register of systematic reviews (PROSPERO) (CRD 42,020,167,307).

### Data source

We searched Pubmed, Embase, the Cochrane Central Register of Controlled Trials, and ClinicalTrials.gov databases, where data was updated to Feb 20, 2020. The top search terms include the following: axitinib, cabozantinib, lenvatinib, nintedanib, pazopanib, regorafenib, sorafenib, sunitinib, vandetanib, and randomized controlled trials (RCT). Searches were performed using Medical Subject Headings (MeSH) terms and free keywords.

We also searched abstracts from the American Society of Clinical Oncology (http://asco.org/ASCO), the European Society of Medical Oncology (http://www.esmo.org/ESMO), and FDA for the period between 2004 and 2020.

### Study selection

The studies’ inclusion criteria were:

1. Phase II and Phase III RCTs involving adult patients with solid tumors.
2. Trials that reported the cardiovascular toxicity of VEGFR-TKI (axitinib, cabozantinib, lenvatinib, nintedanib, pazopanib, regorafenib, sorafenib, sunitinib, vandetanib) versus placebo or one another of adult patients were included.
3. Trials’ safety data of cardiovascular events and sample sizes were available.
4. Trials that reported on at least one of the following clinical outcome measures:

(1) Cardiac disorders: atrial fibrillation; atrial flutter; atrioventricular block; cardiac arrest; conduction disorder; heart failure; left ventricular systolic dysfunction; myocardial infraction; myocarditis; congestive heart failure; cardiac ischemia; fatal cardiovascular incidences; QT prolongation; arrhythmias; (2) hypertension; severe hypertension(graded 3 or higher); (3) cardiovascular-related injuries include: above cardiac disorders, hypertension, arterial thromboembolic events, and venous thrombosis. The outcomes of cardiovascular adverse effects are all defined and graded by the National Cancer Institute (NCI), the Common Terminology Criteria for Adverse Events (CTCAE).

The exclusion criteria were**:**

1. Abstracts, reviews, nonrandomized studies, animal and in vitro studies, meta-analyses, case reports, and subgroup analysis studies.
2. Studies with single-arm VEGFR-TKI treatment include chemotherapy, radiotherapy, or transarterial chemoembolization (TACE); comparable treatment group is no therapy, observation, or best supportive care only; trials designed crossover.
3. Studies sample, including less than 30 subjects.
4. Elderly or pediatric population studies.
5. Studies that did not report effective outcome measures.
6. Unpublished Studies.

We applied no restriction on medication’s dose and study’s publication language.

### Data extraction

Study details were extracted independently by two authors, including the following items: name of the first author, publication year, phase, tumor type, characteristics of the study population (sample size, age, and gender distribution), treatments’ details (previous treatment, therapeutic measures of experimental group and control group, median duration treatment time), clinical-trials.gov registration number and funding source. Patients’ primary cardiovascular conditions, cardiac-related risks (such as hypertension, tobacco use, diabetes, and so on), and adverse occurrences’ defining criterion were also be extracted. The primary outcome was adverse cardiovascular effects (All grades). The second result included serious cardiovascular injuries (Grade 3 or higher), hypertension, serious hypertension (Grade 3 or higher) and cardiac harm. The outcome measure was assessed independently. For the duplicate or subgroup studies, the most recent and complete data were extracted. We also tried to contact study authors to supplement the essential missing data.

### Risk of bias assessment

Two reviewers independently assessed the trials’ risk bias according to the Cochrane Risk of Bias Tool, and the following aspects were evaluated: (1) random sequence generation (selection bias); (2) allocation concealment (selection bias); (3) blinding of participants and personnel (performance bias); (4) blinding of outcome assessment (detection bias); (5) incomplete outcome data (attrition bias); (6) selective reporting (reporting bias) and (7) other bias. Each aspect was evaluated as “high risk,” “unclear risk,” or “low risk.”

### Data synthesis and analysis

We performed meta-analysis using RevMan5.3 to evaluate the direct comparisons’ heterogeneity, calculate OR, and 95% CI. Heterogeneity was tested by the *I*^2^ statistic and Chi-square test *P* value. If *I*^2^ < 50% or Chi-square test *P* < 0.10, the heterogeneity is low, the fixed effects model will be used. Otherwise, high heterogeneity is considered, and the random-effects model will be used. Subgroup analysis and sensitivity analysis will be performed if necessary.

Network analysis was conducted in Stata 15.0 and GeMTC 0.14.3 (Generate Mixed Treatment Comparisons). We checked for inconsistency between all direct and indirect evidence to compare different VEGFR-TKIs’ treatments in terms of cardiovascular hazard and calculated OR along with corresponding 95% credible interval (CrI) in a Bayesian frame using Markov Chain Monte Carlo (MCMC) simulation random-effects model.

The parameters of GeMTC were set as follows: the variance scaling factor is 2.5; the number of simulation iterations is set to 50,000; turning iterations is set to 20,000; the thinning interval is 10; the number of chains is four and inference samples are 10,000. The convergence of the model evaluated the potential scale reduced factor (PSRF). If PSRF is close to 1, the convergence is considered favorable, the consistency of the homogeneity model would be considered reliable enough for follow-up analysis. The “node-splitting” method was assessed consistency between direct and indirect comparisons. The inconsistency test was evaluated according to Bayesian *P* values (*P* < 0.05 is considered to be of significant inconsistency; (Dias et al. 2010).

We evaluated the transitivity of indirect comparisons underlying network meta-analysis by comparing the trials’ clinical and methodological similarity (the trial’s design, the outcome’s assessment, and the participant’s baseline characteristics).

## Results

### Study selection and characteristics

**Fig. 1:**
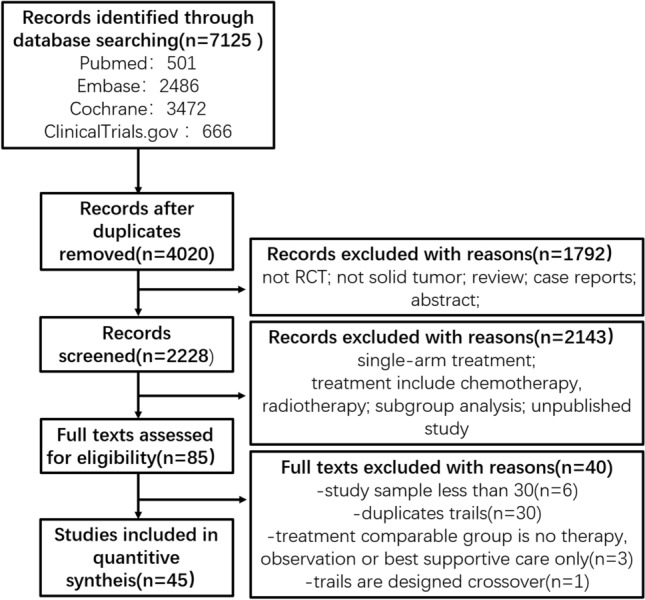
The flow diagram of the study selection

Through searching Pubmed, Embase, the Cochrane Central Register of Controlled Trials, and ClinicalTrials.gov databases, we got a total number of 7125 references. After removing 2105 duplicates, 4020 studies were reserved for further screening, through titles and abstracts selection, 1792 studies were excluded due to unsatisfying the inclusion criteria, through full texts selecting, and 2229 studies were excluded due to unsatisfying the inclusion criteria. We obtained 85 phase II/III RCT published studies. To ensure the meta-analysis’s quality and keep the complete data, we excluded six trials whose sample was including less than 30 subjects and one crossover design trial. Thirty trials were duplicate studies, and we integrated the most recent and complete data of these studies. Three trials whose comparable groups were no therapy, observation, or best supportive care only were also excluded. No additional studies were extracted from ASCO, ESMO, and FDA website. After the above screening, 45 studies were included. The flow diagram for results is shown in Fig. 1, and the PRISMA NMA Checklist of Items are depicted in Supplementary Table S1.

A total of 20,027 patients in 12 phase II and 33 phase III trials of nine FDA-approved VEGFR-TKIs (axitinib, cabozantinib, lenvatinib, nintedanib, pazopanib, regorafenib, sorafenib, sunitinib, vandetanib) were enrolled in the included studies. The main clinical and methodological characteristics of each trial are shown in Table S2. Forty-four trials are two-arm design, and 1 trial is a three-arm design. All Patients’ tumor type is all solid tumor, include hepatocellular carcinoma (HCC), non-small-cell lung cancer (NSCLC), small cell lung cancer (SCLC), renal cell carcinoma (RCC), gastrointestinal stromal tumor (GIST), ovarian cancer (OC), desmoid tumors (DT), colorectal cancer (CRC), thyroid cancer (TC), soft tissue sarcoma (STC) and pancreatic neuroendocrine tumor (PNT). Most of the patients had undergone previous systematic therapy (chemotherapy, targeted drug therapy, radiation, TACE). Only in 4 phase III trials of RCC and 2 phase III trials of HCC, the patients only undergone nephrectomy or not received previous treatment. The mean age of all patients was around 55–65 years. And most of the studies’ proportion of males was more than 40%, except for three trials of ovarian cancer. Most included trials were multicenter, double-blind, randomized design, whereas nine trials were open-label randomized design. Patients’ median duration ranged from 0.9 to 22.2 months, and the trials funding sources almost all were from pharmaceutical companies.

### Quality of included studies

**Fig. 2:**
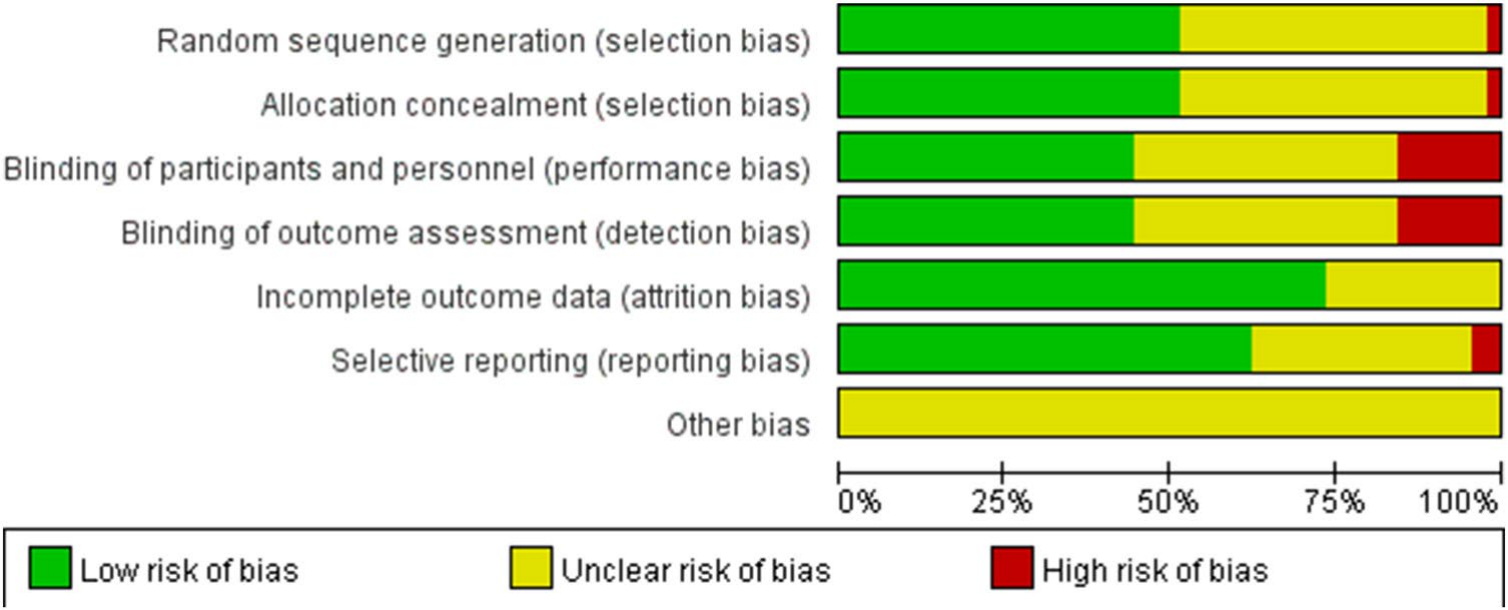
Risk-of-bias graph

Included trials’ bias risk is depicted in Fig. 2. Most studies’ random sequence generation (selection bias) was considered as “unclear risk” or “low risk.” Studies that did not provide details about random sequence generation were recognized as “unclear risk.” The open-label trials were judged as “high risk” both in the performance bias and detection bias. Trials were judged as “unclear risk” of attrition bias because of incomplete outcome-related data in these trials. Most of the trials’ outcome was reported according to the protocol was identified as “low risk” of selective reporting bias. All studies’ other bias was reported as “unclear risk.” Because in some trials, patients’ comorbid cardiac diseases were unknown, and cardiac-related risks such as patients’ smoking status or diabetes status were not reported in most trials (Table S3).

### Network meta-analysis of VEGFR-TKIs’ cardiovascular injuries (all grades).

Initially, 45 trials covering the nine VEGFR-TKIs’ (axitinib, cabozantinib, lenvatinib, nintedanib, pazopanib, regorafenib, sorafenib, sunitinib, vandetanib) cardiovascular circumstances (all grades) were included in the meta-analysis. The total heterogeneity was found high (*I*^2^ = 87%, *P* < 0.00001). The forest plot is presented in Supplement Fig. 1. Further sensitivity analysis did not exclude any single report that could significantly reduce the subgroup analysis heterogeneity based on treatment drugs, clinical phase (phase II or phase III), tumor type, median treatment duration, and trial blind or not were performed. The result shows that treatment drugs were the source of heterogeneity. Most drug subgroup comparisons’ heterogeneity is low (*I*^2^ < 50%, *P* > 0.10), the high heterogeneity was detected in the pazopanib versus the placebo group (*I*^2^ = 75%*, P* = 0.003), sensitivity analysis found the heterogeneity in the pazopanib versus placebo group was caused by Van Der Graaf’s study (2012). So, this study was excluded. The pairwise meta-analysis was showing that cabozantinib, sorafenib, sunitinib, regorafenib, vandetanib, pazopanib, and axitinib were all associated with a higher hazard to cause cardiovascular damage (all grades), as compared to placebo. Also, axitinib was associated with a higher risk of cardiovascular consequence than sorafenib (OR 1.84, 95% CI 1.33–2.53), and the result is displayed in Table 1.

**Table 1:** Pairwise meta-analysis of VEGFR-TKIs’ all grades cardiovascular event

Direct compare	Study number	Total sample	Odds ratio (95% CI)	Method	Heterogeneity
Cabozantinib vs placebo	2	1027	7.38(4.65,11.70)	M–H random	*I*^2^ = 0%(*P* = 0.6)
Suntinib vs placebo	5	2533	4.17 (3.23,5.38)	M–H random	*I*^2^ = 0% (*P* = 0.6)
Sorafenib vs placebo	10	5986	4.65 (3.52,6.15)	M–H random	*I*^2^ = 46% (*P* = 0.05)
Regorafenib vs placebo	6	2050	4.50 (3.39,5.98)	M–H random	*I*^2^ = 0% (*P* = 0.461)
Vandetanib vs placebo	5	1619	11.09 (7.39, 16.63)	M–H random	*I*^2^ = 0% (*P* = 0.42)
Nintedanib vs placebo	2	848	1.42 (0.97,2.07)	M–H random	*I*^2^ = 0% (*P* = 0.36)
Pazopanib vs placebo	4	1568	5.78 (4.50,7.41)	M–H random	*I*^2^ = 0% (*P* = 0.52)
Axitinib vs placebo	2	916	5.96(4.42,8.03)	M–H random	*I*^2^ = 0% (*P* = 0.34)
Sunitinib vs sorafenib	2	2321	1.16(0.94,1.43)	M–H random	*I*^2^ = 0% (*P* = 0.55)
Axitinib vs sorafenib	2	999	1.84(1.33,2.53)	M–H random	*I*^2^ = 22% (*P* = 0.26)
Nintedanib vs sorafenib	2	188	0.61(0.30,1.25)	M–H random	*I*^2^ = 0% (*P* = 0.97)

**Fig. 3:**
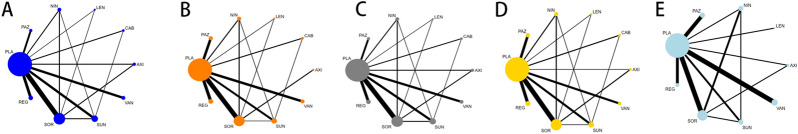
Network diagrams of eligible comparisons. **a** cardiovascular event (All grades), **b** serious cardiovascular event (Grade 3 or higher), hypertension, **d** serious hypertension (Grade 3 or higher), **e** cardiac event. *PLA* placebo, *AXI* axitinib, *CAB* cabozantinib, *LEN* lenvatinib, *NIN* nintedanib, *PAZ* pazopanib, *REG* regorafenib, *SOR* sorafenib, *SUN* sunitinib, *VAN* vandetanib. The size of the points denoted each study’s sample size, the thickness of the lines represented the studies’ number; each line represents the head-to-head comparison

Figure 3a displays the network plots of eligible comparisons for cardiovascular adverse circumstances (All grades). The size of the points denoted each study’s sample size, the thickness of the lines signified the studies’ number, the link between two points indicated direct comparisons.

Network meta-analysis was performed based on the Bayesian frame using an MCMC random-effects model. The head to head comparisons for all grades negative cardiovascular conditions of nine VEGFR-TKIs and placebo was performed under the consistency model (Table 2). Data are ORs (95% CrI) in the column-defining treatment compared with the row-defining treatment. Nine VEGFR-TKIs’ cardiovascular toxicity was all higher than the placebo. Nintedanib exhibited the least cardiovascular toxicity. Lenvatinib was associated with higher cardiovascular toxicity than other VEGFR-TKIs, except for axitinib, vandetanib and cabozantinib. Vandetanib was noted with higher cardiovascular risk than nintedanib, regorafenib, sorafenib, and sunitinib. Axitinib was related to higher cardiovascular toxicity than nintedanib, sorafenib, and sunitinib. The ranking probability was generated based on the MCMC theory for probability evaluation is shown in Supplement Fig. 2. Findings revealed that lenvatinib probably was associated with the highest cardiovascular toxicity among the nine VEGFR-TKIs, followed by vandetanib, cabozantinib, axitinib, pazopanib, sunitinib, sorafenib, regorafenib, and nintedanib. The convergence diagnostic plot drawn according to the Gelman-Rubin-Brooks diagnostic method, the parameter PSRF value is close to 1(Table S4), indicating a favorable convergence. The consistency analysis was performed using the node analysis model. The *P* values of all comparison groups’ direct effect and the indirect effect were higher than 0.05, indicating that the direct results were consistent with the indirect results (Table S5).

**Table 2:** All grades cardiovascular event comparison of nine VEGFR-TKIs

**AXI**									
1.00 (0.48, 2.07)	**CAB**								
0.51 (0.24, 1.08)	0.52 (0.22, 1.19)	**LEN**							
3.66 (1.82, 7.13) ƚ	3.66 (1.63, 7.85) ƚ	7.10 (3.18, 15.47) ƚ	**NIN**						
1.35 (0.69, 2.69)	1.36 (0.63, 2.91)	2.64 (1.18, 5.98) ƚ	0.37 (0.18, 0.79)*	**PAZ**					
8.06 (5.23, 13.02) ƚ	8.16 (4.55, 14.56) ƚ	15.72 (8.46, 30.11) ƚ	2.22 (1.30, 3.86) ƚ	5.94 (3.61, 9.94) ƚ	**PLA**				
1.76 (0.97, 3.36)	1.77 (0.86, 3.74)	3.43 (1.64, 7.53) ƚ	0.49 (0.25, 0.99)*	1.30 (0.67, 2.51)	0.22 (0.14, 0.34)*	**REG**			
1.60 (1.02, 2.51) ƚ	1.61 (0.84, 2.99)	3.11 (1.69, 5.74) ƚ	0.44 (0.26, 0.75)*	1.18 (0.66, 2.06)	0.20 (0.15, 0.25)*	0.91 (0.54, 1.49)	**SOR**		
1.74 (1.03, 3.16)) ƚ	1.76 (0.95, 3.31)	3.40 (1.72, 7.09) ƚ	0.48 (0.27, 0.89)*	1.29 (0.71, 2.40)	0.22 (0.15, 0.32)*	0.99 (0.57, 1.77)	1.10 (0.76, 1.66)	**SUN**	
0.64 (0.31, 1.32)	0.64 (0.29, 1.43)	1.24 (0.53, 2.90)	0.18 (0.08, 0.38)*	0.47 (0.22, 1.00)	0.08 (0.04, 0.14)*	0.36 (0.17, 0.72)*	0.40 (0.21, 0.75)*	0.36 (0.18, 0.70)*	**VAN**

Data presented with ORs (95% CrI) in the column-defining drug compared with the row-defining drug

*OR* odds ratio, *CrI* credible interval, *PLA* placebo, *AXI* axitinib, *CAB* cabozantinib, *LEN* lenvatinib, *NIN* nintedanib, *PAZ* pazopanib, *REG* regorafenib, *SOR* sorafenib, *SUN* sunitinib, *VAN* vandetanib

*ORs more than 1 favors row-defining drug

ƚ ORs less than 1 favors column-defining drug

### Network meta-analysis of VEGFR-TKIs’ cardiovascular severe damage (grade 3 or higher)

Forty-two trials comprising the nine VEGFR-TKIs reported adverse cardiovascular impacts (grade3 or higher). High heterogeneity was found in all included trials (*I*^2^ = 66%, *P* < 0.00001). The forest plot is depicted in Supplement Fig. 3. Further subgroup analysis found that different treatment drugs caused heterogeneity. Subgroup analysis shows high heterogeneity that was identified in the group of cabozantinib versus placebo (*I*^2^ = 61%, *P* = 0.11), regorafenib versus placebo (*I*^2^ = 52%, *P* = 0.07) and axitinib versus sorafenib (*I*^2^ = 93%, *P* = 0.0001). Considering the limited study number of these groups, we included these studies into the network meta-analysis, and the random-effects model was used. The network plots are shown in Fig. 3b. Results of the pairwise meta-analysis of the severe cardiovascular injury showed that sunitinib, sorafenib, vandetanib, nintedanib, and pazopanib, were all associated with a higher hazard to inflict severe cardiovascular toxicity, as compared to placebo; details are displayed in Table 3.

**Table 3:** Pairwise meta-analysis of VEGFR-TKIs’ serious cardiovascular event (grade 3 or high)

Direct compare	Study number	Total sample	Odds ratio (95% CI)	Method	Heterogeneity
Cabozantinib vs placebo	2	1027	10.56 (2.39,46.58)	M–H random	*I*^2^ = 61% (*P* = 0.11)
Suntinib vs placebo	4	1282	6.33 (2.54,15.78)	M–H random	*I*^2^ = 16% (*P* = 0.31)
Sorafenib vs placebo	9	4732	4.97 (3.25,7.59)	M–H random	*I*^2^ = 0% (*P* = 0.69)
Regorafenib vs placebo	6	2050	4.69 (2.29,9.60)	M–H random	*I*^2^ = 52% (*P* = 0.07)
Vandetanib vs placebo	5	1619	8.29 (3.74,18.40)	M–H random	*I*^2^ = 0% (*P* = 0.47)
Nintedanib vs placebo	2	848	6.35 (2.18,18.52)	M–H random	*I*^2^ = 0% (*P* = 0.64)
Pazopanib vs placebo	5	2038	7.03 (4.85,10.18)	M–H random	*I*^2^ = 0% (*P* = 0.89)
Axitinib vs sorafenib	2	999	1.79 (0.10,32.69)	M–H random	*I*^2^ = 93% (*P* = 0.00001)
Nintedanib vs sorafenib	2	188	1.09 (0.29,4.11)	M–H random	*I*^2^ = 0% (*P* = 0.82)

Results of the Bayesian network meta-analysis of the severely harmful cardiovascular occurrence are summarized in Table 4. Lenvatinib was associated with higher harm for the severe injury cardiovascular effects than nintedanib, pazopanib, regorafenib, sorafenib, and sunitinib. The parameter PSRF value is close to 1, indicating the model’s convergence is good (Table S6). The ranking probability is depicted in Supplement Fig. 4, lenvatinib ranked most likely to have the highest injury of cardiovascular toxicity among the nine VEGFR-TKIs, followed by axitinib, the difference between vandetanib, cabozantinib, sorafenib, nintedanib, regorafenib, pazopanib, and sunitinib was not obvious. Most direct results and indirect results did not show significant inconsistency, while in lenvatinib vs. sorafenib and lenvatinib vs. sunitinib group, the *P*-values between direct and indirect results were less than 0.05, indicating the inconsistency between the direct studies and indirect studies (Table S7).

**Table 4:** Serious cardiovascular event comparison of nine VEGFR-TKIs

**AXI**									
1.76 (0.36, 9.27)	**CAB**								
0.42 (0.09, 2.09)	0.24 (0.05, 1.02)	**LEN**							
2.97 (0.67, 14.63)	1.71 (0.40, 7.05)	7.10 (1.60, 31.96) ƚ	**NIN**						
3.10 (0.71, 14.98)	1.77 (0.47, 7.17)	7.37 (1.81, 34.94) ƚ	1.05 (0.28, 4.17)	**PAZ**					
20.00 (6.00, 77.34) ƚ	11.40 (4.14, 33.87) ƚ	47.29 (15.29, 163.22) ƚ	6.71 (2.41, 20.69) ƚ	6.47 (2.83, 15.08) ƚ	**PLA**				
3.90 (0.93, 17.80)	2.21 (0.61, 8.03)	9.16 (2.41, 38.42) ƚ	1.30 (0.35, 4.89)	1.24 (0.41, 3.74)	0.19 (0.09, 0.39)*	**REG**			
2.68 (0.95, 8.72)	1.54 (0.50, 4.93)	6.38 (2.26, 20.43) ƚ	0.91 (0.31, 2.75)	0.87 (0.31, 2.44)	0.14 (0.07, 0.25)*	0.70 (0.27, 1.81)	**SOR**		
2.78 (0.73, 11.51)	1.60 (0.52, 4.72)	6.64 (2.21, 21.44) ƚ	0.93 (0.30, 3.01)	0.90 (0.28, 2.78)	0.14 (0.06, 0.28)*	0.73 (0.25, 2.04)	1.03 (0.46, 2.27)	**SUN**	
1.50 (0.26, 8.14)	0.85 (0.17, 3.81)	3.59 (0.65, 18.07)	0.50 (0.10, 2.38)	0.47 (0.11, 1.83)	0.07 (0.02, 0.21)*	0.39 (0.09, 1.37)	0.55 (0.14, 1.87)	0.53 (0.12, 1.95)	**VAN**

Data presented with ORs (95% CrI) in the column-defining drug compared with the row-defining drug

*OR* odds ratio, *CrI* credible interval, *PLA* placebo, *AXI* axitinib, *CAB* cabozantinib, *LEN* lenvatinib, *NIN* nintedanib, *PAZ* pazopanib, *REG* regorafenib, *SOR* sorafenib, *SUN* sunitinib, *VAN* vandetanib

*ORs more than 1 favors row-defining drug

ƚ ORs less than 1 favors column-defining drug

### Network meta-analysis of VEGFR-TKIs’ hypertension (all grades)

All grades of hypertension conditions were reported in the 45 trials, covering the nine VEGFR-TKIs. All included studies showed high heterogeneity (*I*^2^ = 84%, *P* = 0.00001). The forest plot is illustrated in Supplement Fig. 5. Further sensitivity analysis did not find a significant result. Subgroup analysis based on drugs found the high heterogeneity in the pazopanib versus the placebo group (*I*^2^ = 70%, *P* = 0.009). Sensitivity analysis found the heterogeneity was caused by Van Der Graaf’s study (2012). Thus, this last study was excluded from the network meta-analysis. The network plot is exhibited in Fig. 3c. The pairwise meta-analysis showed that cabozantinib, sunitinib, sorafenib, regorafenib, vandetanib, pazopanib, and axitinib were all associated with a higher risk of all grades of hypertension conditions when compared to placebo. Axitinib elicited a higher hypertension risk than sorafenib (OR 1.82, 95% CI 1.34––2.45); details are shown in Table 5.

**Table 5:** Pairwise meta-analysis of VEGFR-TKIs’ all grades hypertension

Direct compare	Study number	Total sample	Odds ratio (95% CI)	Method	Heterogeneity
Cabozantinib vs placebo	2	1027	7.42 (4.54,12.13)	M–H random	*I*^2^ = 0% (*P* = 0.45)
Suntinib vs placebo	5	2533	4.74 (3.60,6.24)	M–H random	*I*^2^ = 0% (*P* = 0.76)
Sorafenib vs placebo	10	5986	4.89 (3.72,6.43)	M–H random	*I*^2^ = 40% (*P* = 0.09)
Regorafenib vs placebo	6	2050	5.16 (3.80,7.01)	M–H random	*I*^2^ = 0% (*P* = 0.99)
Vandetanib vs placebo	5	1619	10.56 (6.56, 17.01)	M–H random	*I*^2^ = 0% (*P* = 0.97)
Nintedanib vs placebo	2	848	1.40 (0.95,2.06)	M–H random	*I*^2^ = 0% (*P* = 0.42)
Pazopanib vs placebo	4	1568	7.18 (4.82, 10.70)	M–H random	*I*^2^ = 0% (*P* = 0.91)
Axitinib vs placebo	2	916	5.83 (4.32,7.86)	M–H random	*I*^2^ = 0% (*P* = 0.44)
Sunitinib vs sorafenib	2	2321	1.14 (0.92,1.41)	M–H random	*I*^2^ = 0% (*P* = 0.41)
Axitinib vs sorafenib	2	999	1.82 (1.34,2.45)	M–H random	*I*^2^ = 15% (*P* = 0.28)
Nintedanib vs sorafenib	2	188	0.93 (0.40,2.18)	M–H random	*I*^2^ = 0% (*P* = 0.34)

Results of the Bayesian network meta-analysis of hypertension effects are depicted in Table 6. Lenvatinib’s hypertension risk was higher than nintedanib, pazopanib, regorafenib, sorafenib, and sunitinib. Vandetanib’s hypertension harm was more elevated than nintedanib, pazopanib, regorafenib, sorafenib, sunitinib. Axitinib’s hypertension risk was higher than nintedanib and sorafenib. The parameter PSRF value is close to 1 indicated the model’s convergence is adequated (Table S8). The ranking probability is displayed in Supplement Fig. 6. Lenvatinib had the most significant probability of provoking hypertension, followed by vandetanib. cabozantinib, axitinib, pazopanib, sorafenib, sunitinib, regorafenib, and nintedanib. The direct comparison and indirect comparison did not show significant inconsistency (Table S9).

**Table 6:** All grade hypertension comparison of nine VEGFR-TKIs

**AXI**									
0.92 (0.45, 1.87)	**CAB**								
0.53 (0.25, 1.04)	0.57 (0.25, 1.26)	**LEN**							
2.78 (1.31, 5.49) ƚ	3.01 (1.29, 6.66) ƚ	5.29 (2.30, 11.81) ƚ	**NIN**						
1.52 (0.82, 2.96)	1.66 (0.79, 3.60)	2.89 (1.40, 6.43) ƚ	0.55 (0.27, 1.24)	**PAZ**					
7.87 (5.20, 12.23) ƚ	8.58 (4.85, 15.57) ƚ	14.91 (8.64, 27.74) ƚ	2.85 (1.61, 5.36) ƚ	5.18 (3.19, 8.29) ƚ	**PLA**				
1.50 (0.82, 2.72)	1.62 (0.80, 3.33)	2.83 (1.41, 5.96) ƚ	0.54 (0.27, 1.16)	0.98 (0.51, 1.82)	0.19 (0.12, 0.29)*	**REG**			
1.54 (1.01, 2.35) ƚ	1.67 (0.91, 3.15)	2.92 (1.69, 5.31) ƚ	0.56 (0.31, 1.04)	1.01 (0.58, 1.70)	0.20 (0.15, 0.25))*	1.03 (0.63, 1.68)	**SOR**		
1.56 (0.94, 2.66)	1.69 (0.92, 3.22)	2.94 (1.59, 5.92) ƚ	0.56 (0.30, 1.12)	1.02 (0.56, 1.82)	0.20 (0.14, 0.28)*	1.04 (0.61, 1.79)	1.01 (0.71, 1.47)	**SUN**	
0.69 (0.33, 1.43)	0.74 (0.33, 1.73)	1.30 (0.58, 3.05)	0.25 (0.11, 0.60)*	0.45 (0.21, 0.95)*	0.09 (0.05, 0.16)*	0.54 (0.20, 1.39)*	0.45 (0.23, 0.84)*	0.44 (0.22, 0.87)*	**VAN**

Data presented with ORs (95% CrI) in the column-defining drug compared with the row-defining drug

*OR* odds ratio, *CrI* credible interval, *PLA* placebo, *AXI* axitinib, *CAB* cabozantinib, *LEN* lenvatinib, *NIN* nintedanib, *PAZ* pazopanib, *REG* regorafenib, *SOR* sorafenib, *SUN* sunitinib, *VAN* vandetanib

*ORs more than 1 favors row-defining drug

ƚ: ORs less than 1 favors column-defining drug

### Network meta-analysis of VEGFR-TKIs’ severe hypertension (grade 3 or higher)

Serious hypertension conditions were reported in 42 trials, covering all the nine VEGFR-TKIs. For all included trials, high heterogeneity was found (*I*^2^ = 63%, *P* < 0.00001). The forest plot is exhibited in Supplement Fig. 7. Further subgroup analysis based on individual drugs found the high heterogeneity in the axitinib versus sorafenib group (*I*^2^ = 82%, *P* = 0.02). The network plot is displayed in Fig. 3d. A pairwise meta-analysis of severe hypertension showed that cabozantinib, sunitinib, sorafenib, regorafenib, vandetanib, nintedanib and pazopanib were all associated with a higher risk of serious injury hypertension events, as compared to the placebo control group. The details of these results are shown in Table 7.

**Table 7:** Pairwise meta-analysis of VEGFR-TKIs’ serious hypertension (grade 3 or higher)

Direct compare	Study Number	Total Sample	Odds Ratio (95% CI)	Method	Heterogeneity
Cabozantinib vs placebo	2	1027	17.02 (5.34,54.26)	M–H random	*I*^2^ = 0% (*P* = 0.52)
Suntinib vs placebo	4	1282	8.82 (3.43,22.70)	M–H random	*I*^2^ = 0% (*P* = 0.61)
Sorafenib vs placebo	9	4732	5.20 (3.22,8.39)	M–H random	*I*^2^ = 0% (*P* = 0.74)
Regorafenib vs placebo	6	2050	4.42 (2.29,8.54)	M–H random	*I*^2^ = 38% (*P* = 0.15)
Vandetanib vs placebo	4	1474	4.22 (1.74,10.23)	M–H random	*I*^2^ = 0% (*P* = 0.49)
Nintedanib vs placebo	2	848	5.99 (1.91,18.78)	M–H random	*I*^2^ = 0% (*P* = 0.89)
Pazopanib vs placebo	5	2038	6.89 (4.72, 10.04)	M–H random	*I*^2^ = 0% (*P* = 0.91)
Axitinib vs sorafenib	2	999	3.87 (0.36,41.97)	M–H random	*I*^2^ = 82% (*P* = 0.02)
Nintedanib vs sorafenib	2	188	1.90 (0.30,11.92)	M–H random	*I*^2^ = 0% (*P* = 0.88)

The findings of the Bayesian network meta-analysis are displayed in Table 8. Sorafenib was superior to axitinib, cabozantinib, and lenvatinib in terms of serious hypertension hazard. The parameter PSRF value is close to 1, which indicated the model’s convergence is acceptable (Table S10). The ranking probability is depicted in Supplement Fig. 8. The nine agent’s severe hypertension risk was probably similar. Direct results and indirect results did not present inconsistencies (Table S11).

**Table 8:** Serious hypertension comparison of nine VEGFR-TKIs

**AXI**									
0.91 (0.24, 4.04)	**CAB**								
0.85 (0.27, 3.30)	0.95 (0.22, 3.92)	**LEN**							
2.12 (0.49, 9.37)	2.35 (0.47, 10.30)	2.49 (0.55, 10.42)	**NIN**						
2.60 (0.85, 10.58)	2.91 (0.84, 11.18)	3.06 (0.97, 11.30)	1.24 (0.35, 5.41)	**PAZ**					
16.62 (6.90, 53.02) ƚ	18.33 (6.77, 56.72) ƚ	19.47 (7.94, 54.59) ƚ	7.87 (2.76, 28.34) ƚ	6.37 (3.02, 13.30) ƚ	**PLA**				
2.21 (0.73, 8.38)	2.42 (0.71, 9.23)	2.56 (0.82, 8.78)	1.04 (0.29, 4.55)	0.84 (0.29, 2.29)	0.13 (0.06, 0.27)*	**REG**			
2.76 (1.28, 7.84) ƚ	3.02 (1.02, 9.92) ƚ	3.23 (1.33, 8.53) ƚ	1.32 (0.44, 4.65)	1.06 (0.42, 2.53)	0.17 (0.10, 0.27)*	1.26 (0.52, 2.97)	**SOR**		
1.31 (0.43, 4.74)	1.46 (0.50, 4.11)	1.53 (0.46, 5.16)	0.63 (0.18, 2.39)	0.50 (0.16, 1.42)	0.08 (0.03, 0.17)*	0.60 (0.20, 1.69)	0.47 (0.20, 1.05)	**SUN**	
2.35 (0.50, 11.40)	2.58 (0.50, 12.28)	2.72 (0.56, 11.93)	1.14 (0.21, 5.62)	0.89 (0.20, 3.21)	0.14 (0.04, 0.40)*	1.05 (0.25, 3.89)	0.84 (0.21, 2.74)	1.77 (0.40, 6.94)	**VAN**

Data presented with ORs (95% CrI) in the column-defining drug compared with the row-defining drug

*OR* odds ratio, *CrI* credible interval, *PLA* placebo, *AXI* axitinib, *CAB* cabozantinib, *LEN* lenvatinib, *NIN* nintedanib, *PAZ* pazopanib, *REG* regorafenib, *SOR* sorafenib, *SUN* sunitinib, *VAN* vandetanib

*ORs more than 1 favors row-defining drug

ƚ: ORs less than 1 favors column-defining drug

### Network meta-analysis of VEGFR-TKIs’ cardiac repercussions

The cardiac incidences were reported in 25 trials, covering eight VEGFR-TKIs (axitinib, lenvatinib, nintedanib, pazopanib, regorafenib, sorafenib, sunitinib, vandetanib). The total heterogeneity of all included trials is moderate (*I*^2^ = 48%, *P* = 0.003). The forest plot is exhibited in Supplement Fig. 9. Subgroup based on treatment drugs found the moderate heterogeneity in the sorafenib versus the placebo group (*I*^2^ = 40%, *P* = 0.16) and regorafenib versus the placebo group (*I*^2^ = 43%, *P* = 0.18). Results of a pairwise meta-analysis of the cardiac occurrences found that vandetanib and pazopanib were associated with higher cardiac harm than the placebo group (Table 9).

**Table 9:** Pairwise meta-analysis of VEGFR-TKIs’ cardiac event

Direct compare	Study number	Total sample	Odds ratio (95% CI)	Method	Heterogeneity
Suntinib vs placebo	2	1416	1.37(0.65,2.88)	M–H random	*I*^2^ = 0% (*P* = 0.38)
Sorafenib vs placebo	5	4278	2.12 (0.86,5.19)	M–H Random	*I*^2^ = 40% (*P* = 0.16)
Regorafenib vs placebo	3	583	1.16 (0.24,5.63)	M–H random	*I*^2^ = 43% (*P* = 0.18)
Vandetanib vs placebo	5	1619	15.01 (4.66, 48.37)	M–H random	*I*^2^ = 0% (*P* = 0.92)
Pazopanib vs placebo	4	1943	6.67(1.98,22.51)	M–H random	*I*^2^ = 0% (*P* = 0.73)
Nintedanib vs sorafenib	2	188	0.31(0.04,2.56)	M–H random	*I*^2^ = 0% (*P* = 0.62)

Figure 3e presents the network plots of eligible comparisons. In the convergence test, we found the PSRF value of lenvatinib versus placebo is 1.22, which denoted an unsatisfactory convergence. So, we did not include the data of lenvatinib. The convergence test of other agents presents a good convergence (Table S12). Network meta-analysis results revealed that vandetanib was associated with a higher risk for the adverse cardiac effects than axitinib, nintedanib, regorafenib, and sorafenib sunitinib, and placebo; the details are included in Table 10. Pazopanib was associated with a higher risk for cardiac incidences as compared with nintedanib and placebo. In the harm ranking possibility shown in Supplement Fig. 10, vandetanib graded to be the most likely to have the highest hazard for the unfavorable cardiac occurrences among VEGFR-TKIs, followed by pazopanib, axitinib, sorafenib, sunitinib. In contrast, regorafenib and nintedanib may do not exhibited an increased risk of a cardiac injury. Inconsistency between direct and indirect estimates from the node splitting analysis did not show significant differences in comparisons (Table S13).

**Table 10:** Cardiac event comparison of seven VEGFR-TKIs

**AXI**							
3.35 (0.38, 47.13)	**NIN**						
0.28 (0.02, 4.46)	0.08 (0.01, 0.54)*	**PAZ**					
2.78 (0.40, 30.78)	0.83 (0.26, 2.74)	10.10 (2.15, 78.96)*	**PLA**				
1.88 (0.14, 33.08)	0.56 (0.08, 4.01)	6.98 (0.80, 86.82)	0.68 (0.14, 3.07)	**REG**			
1.37 (0.19, 13.53)	0.43 (0.10, 1.34)	5.10 (0.81, 40.38)	0.51 (0.18, 1.04)	0.73 (0.11, 4.22)	**SOR**		
1.41 (0.15, 17.30)	0.43 (0.11, 1.40)	5.18 (0.74, 48.21)	0.52 (0.15, 1.34)	0.76 (0.10, 4.88)	1.00 (0.34, 3.32)	**SUN**	
0.03 (0.00, 0.56)*	0.01 (0.00, 0.08)*	0.10 (0.00, 1.70)	0.01 (0.00, 0.08)*	0.01 (0.00, 0.22)*	0.02 (0.00, 0.18)*	0.02 (0.00, 0.19)*	**VAN**

Data presented with ORs (95% CrI) in the column-defining drug compared with the row-defining drug

*OR* odds ratio, *CrI* credible interval, *PLA* placebo, *AXI* axitinib, *CAB* cabozantinib, *LEN* lenvatinib, *NIN* nintedanib, *PAZ* pazopanib, *REG* regorafenib, *SOR* sorafenib, *SUN* sunitinib, *VAN* vandetanib

*ORs more than 1 favors row-defining drug

ƚ: ORs less than 1 favors column-defining drug

### Rank probabilities

**Fig. 4:**
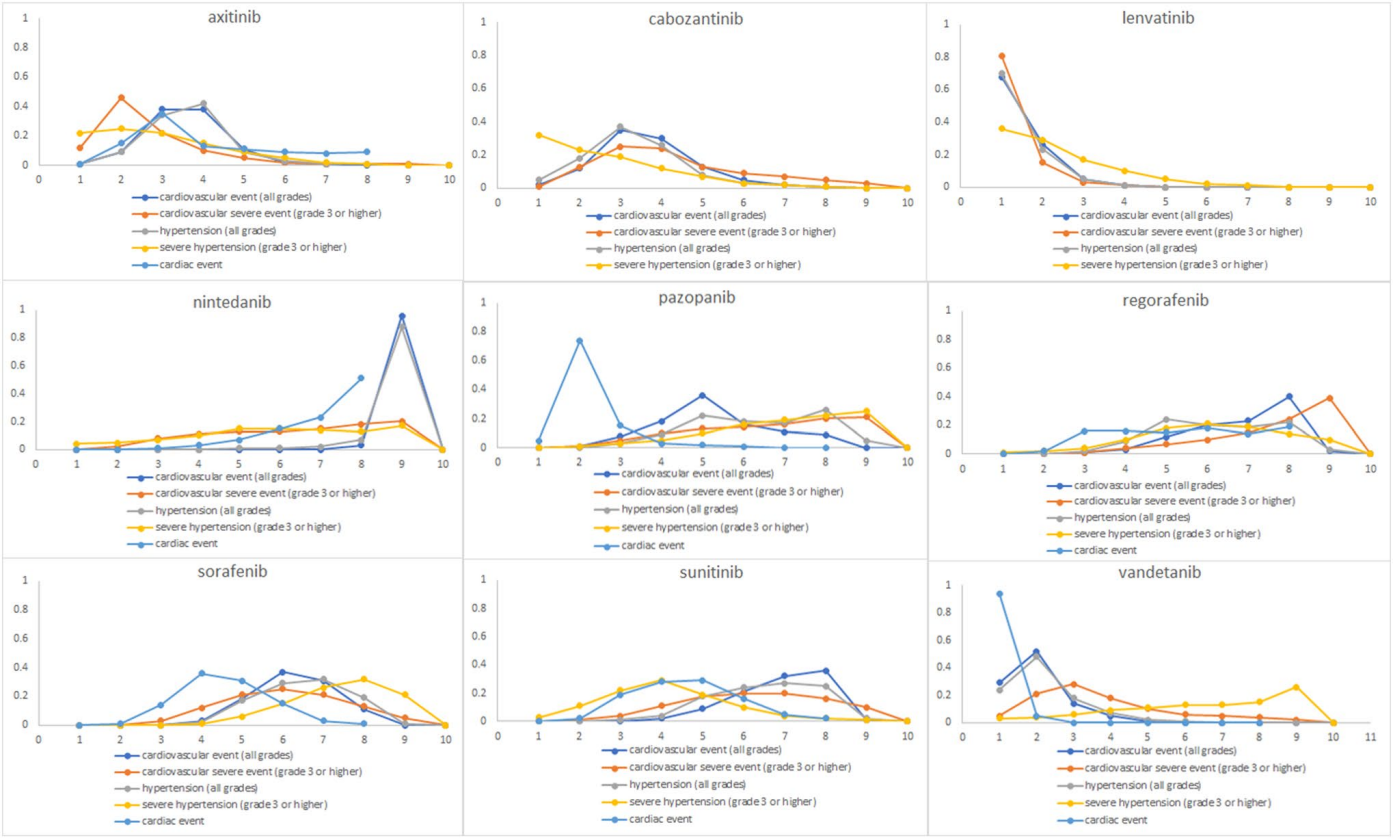
The adverse cardiovascular occurrences gradient induced by the nine VEGFR-TKIs; grade 3 or higher, hypertension, severe hypertension, and cardiac circumstances

We did a summary of each VEGFR-TKIs’ adverse cardiovascular circumstances (all grades), severe cardiovascular event (grade 3 or higher), hypertension (all grades), severe hypertension (grade 3 or higher), and cardiac incidents (Fig. 4). We concluded that nintedanib might be related to less cardiovascular risk (including all grades cardiovascular effects, all grades hypertension, and cardiac event) among the nine VEGFR-TKIs studied. The next less cardiotoxic drug was regorafenib. Lenvatinib was associated with a higher occurrence of cardiovascular disorders and hypertension (both all grades and severe). Vandetanib and pazopanib induced notorious cardiac toxicity. At the same time, axitinib was associated with a higher risk of severe cardiovascular damage.

## Discussion

Multiple VEGFR-TKIs demonstrated effectiveness in cancer patients eliminating solid tumors, either as a single agent or using combinatorial treatment strategies. The FDA had approved nine VEGFR-TKIs for different types of solid tumors. However, cardiovascular harm was found in these drugs, and head-to-head randomized controlled trials of the nine VEGFR-TKIs are lacking. Thus, it is hard for clinicians to choose a safe drug for solid tumor patients, with less cardiovascular toxicity among all of them. The network meta-analysis’s appearance offset the limitation of a pairwise meta-analysis on indirect comparisons, and the Bayesian NMA could provide a rank for different comparable treatment of efficacy and safety (Lumley 2002). More clinicians and specialists in evidence-based medicine search to find useful suggestions from NMA may provide useful safety indicators for the clinical medication in recent years (Cipriani et al. 2018; Liu et al. 2017b; Palmer et al. 2014).

In this Bayesian network meta-analysis, we investigated the cardiovascular toxicity associated with nine FDA-approved VEGFR-TKIs, implicating cardiovascular events risk (all grades and grade 3 or higher), hypertension (all grades and grade 3 or higher), and cardiac injuries. Findings indicated that the nine VEGFR-TKIs involved in this study were associated with different degrees of cardiovascular risk than the placebo control group.

The nine drugs induced different degrees of cardiovascular-related toxicity. For instance, nintedanib may be associated with less cardiovascular risk (including all grades cardiovascular disorders, all grades hypertension, and cardiac event) among all nine VEGFR-TKIs. Lenvatinib was related to a higher occurrence of cardiovascular injury and hypertension (all severity grades). The risk of severe cardiovascular and severe hypertension risk was probably similar among nine agents. Vandetanib ranked most likely to have the highest risk for cardiotoxicity, followed by pazopanib, axitinib, sorafenib, sunitinib. Regorafenib and nintedanib did not exhibit an increased risk of cardiac harm.

## The strengths of our network meta-analysis

We designed this network meta-analysis according to standardized PRISMA NMA principles and conducted it carefully to minimize errors and ensure the validity of findings from all relevant studies identified. To our knowledge, this is the first network meta-analysis to compare and rank the cardiovascular hazard of VEGFR-TKIs. We included all available RCTs of nine approved VEGFR-TKIs and combined them with pairwise meta-analysis and network meta-analysis. The details of each implicated study were recorded, including the characteristics of involved trials, also patients’ primary cardiovascular conditions, cardiac-related risks (such as hypertension, tobacco use, diabetes), and adverse events’ defining criteria were extracted. Strict measures were undertaken to prevent bias. Nine agents’ safety data of cardiovascular events (all grades), serious cardiovascular events (Grade 3 or higher), hypertension, serious hypertension (Grade 3 or higher) and cardiac events were extracted individually. Consider the potential heterogeneity, random-effects modeling was used both in meta-analysis and network meta-analysis. A pairwise meta-analysis of included trials was tested at first. Sensitivity analysis and subgroup analysis were used to found the source of heterogeneity. The group analyzed the treatment drugs, clinical phase (phase II or phase III), tumor type, median treatment duration, and trial blind or not. The trial presenting with high heterogeneity in subgroups was not included in the following NMA. Moreover, the Bayesian approach was used to perform the NMA, which could be thought more flexible, and results are more clinically interpretable than the frequentist approach (Lu and Ades 2009). The node-splitting method revealed that most direct and indirect results did not present significant inconsistencies. The results demonstrated some similarities with the previous studies and meta-analysis. Such as lenvatinib’s potential higher cardiovascular and hypertension risk than other VEGFR-TKIs (Oba et al. 2020). Axitinib was associated with a higher risk of hypertension event than sunitinib (Bæk et al. 2019). And vandetanib’s higher cardiac toxicity (Ton et al. 2013). Furthermore, this NMA provided the ranking possibility to induce cardiovascular damage (all grades) of nine commonly used VEGFR-TKIs’, severe cardiovascular event (Grade 3 or higher), hypertension, serious hypertension (Grade 3 or higher) and cardiac event risk.

### This NMA has clinical implications

The VEGFR-TKIs are promising drugs for solid tumors’ treatment. The nine FDA-approved VEGFR-TKIs are used on different solid tumors, especially in HCC, RCC, and TC. Some VEGFR-TKIs’ efficacy was similar (Kourie et al. 2018; Manz et al. 2020); safety and secondary adverse effects may the key determining factors for drug selection. Hypertension was found in most VEGFR-TKIs; cardiac events, and the arterial thrombosis or venous thrombosis incidents were also reported in some VEGFR-TKIs. At this point, it is hard to elucidate the mechanism of action, causing the adverse effect cardiovascular of the VEGFR-TKIs because most of them own more than one and unique target. The reviewed nine VEGFR-TKIs were associated with higher cardiovascular harm than placebo from previous pairwise meta-analysis, but it was unknown the individual degree for each drugs’ cardiovascular risk. Nevertheless, this is important in the clinic, especially for patients with preexistence cardiovascular disease conditions; more safe drugs are essential. Our study pooled and ranked the cardiovascular risk of these drugs using the data from individual RCTs. These findings may be useful to clinicians in their decisions on which medicine to choose.

### There are also some limitations to our study

First, this study included a limited number of trials, and the comparison RCTs between VEGFR-TKIs are also limited; most of the evidence is from the VEGFR-TKIs and placebo’s evaluation. Second, there are still missing some outcome data even though we tried to contact the corresponding authors and pharmaceutical companies. Moreover, it is hard to acquire an individual patient’s cardiovascular adverse effects records when exposed to VEGFR-TKIs or other drugs. Third, though the total sample of our meta-analysis is enough for some comparison group, however, in some instances, the sample size is still lacking sufficient subjects. Some estimated results of the network meta-analysis relied on indirect comparisons. Fourth, the cardiac-related factors (such as smoking, diabetes, or prevalent cardiac conditions) were not reported in most included studies, which may cause heterogeneity between studies. And it is common for oncology trials to report adverse events (AEs) irrespective of causality, which may cause inaccurate attribution of AEs.

## Conclusions

This network meta-analysis (NMA) was the first study to provide a gradient of the possibility of using nine VEGFR-TKIs-FDA-approved medicines as anticancer drugs on patients with solid tumors, focusing on the cardiotoxicity risk damage and analyzed under the Bayesian theory frame. Findings indicated that lenvatinib revealed the greatest probability of provoking all grades of cardiovascular incidents and hypertension, followed by vandetanib, cabozantinib, axitinib, pazopanib, sorafenib, sunitinib, regorafenib, and nintedanib. In contrast, regorafenib and nintedanib did not exhibit an apparent increase in cardiotoxicity risk occurrence. Thus, our findings may provide useful information for clinical practice guideline implementation strategies selecting the adequate VEGFR-TKIs and understanding the cardiovascular toxicity inflicted by the VEGFR-TKIs.

## Supplementary Information

Below is the link to the electronic supplementary material.

Supplementary file1 (DOCX 25 KB)

Supplementary file2 (DOCX 51 KB)

Supplementary file3 (DOCX 19 KB)

Supplementary file4 (DOCX 34 KB)

Supplementary file5 (PDF 1537 KB)

Supplementary file6 (PNG 20 KB)

Supplementary file7 (PDF 1330 KB)

Supplementary file8 (PDF 1522 KB)

Supplementary file9 (PNG 20 KB)

Supplementary file10 (PDF 1362 KB)

Supplementary file11 (PNG 20 KB)

Supplementary file12 (PDF 935 KB)

Supplementary file13 (PNG 18 KB)
