# Supplementary material for: Comparative evaluation of cardiovascular risks among nine FDA-approved VEGFR-TKIs in patients with solid tumors: a Bayesian network analysis of randomized controlled trials
Source: J Cancer Res Clin Oncol. 2021 Mar 16;147(8):2407–20. doi: 10.1007/s00432-021-03521-w (PMC8236482; doi:10.1007/s00432-021-03521-w)
Supplement: Supplementary file 2 — Supplementary file2 (DOCX 51 KB) [file 432_2021_3521_MOESM2_ESM.docx]

**Supplement Table 2** Characteristics of involved trials

| **Study ID** | **Phase** | **Tumor type** | **Previous treatment** | **No. of patients enrolled** | **Treatment arm** | **Evaluable patients per arm** | **Median age (range) years** | **Male（%）** | **Clinical-Trials.gov registration** | **Jadad score** | **Center** | **Funding** | **Median treatment duration** |
| --- | --- | --- | --- | --- | --- | --- | --- | --- | --- | --- | --- | --- | --- |
| Abou-Alfa | phase III | HCC | previous sorafenib | 704 | cabozantinib 60mg qd | n=467 | 64 (22–86) | 81.0% | NCT01908426 | 6 | multicentre | Exelixis | 3.8months |
| 2018 |  |  |  |  | placebo | n=237 | 64 (24–86) | 85.0% |  |  |  |  | 2.0months |
| Ahn | phase III | NSCLC | chemotherapy | 117 | vandetanib 300mg qd | n=75 | 61 (33–76) | 62.7% | NCT00777179 | 3 | multicentre | AstraZeneca | 59days |
| 2013 |  |  |  |  | placebo | n=42 | 60.5 (29–70) | 66.7% |  |  |  |  | 54days |
| Arnold | phase II | SCLC | chemotherapy | 105 | vandetanib 300mg qd | n=52 | 56.9 | 50.9% | NR | 4 | NR | AstraZeneca | 7weeks |
| 2007 |  |  |  |  | placebo | n=53 | 62.4 | 57.4% |  |  |  |  | 12weeks |
| Baggstrom | phase III | NSCLC | chemotherapy | 198 | sunitinib 37.5mg qd | n=99 | 65(25-84) | 53.8% | NCT00693992 | 4 | USA | Pfizer | NR |
| 2017 |  |  |  |  | placebo | n=99 | 67(44-89) | 57.7% |  |  | multicentre |  | NR |
| Brose | phase III | TC | radiotherapy | 416 | sorafenib 400mg bid | n=207 | 63 (24–82) | 50.2% | NCT00984282 | 7 | multicentre | Bayer | 10.6months |
| 2014 |  |  |  |  | placebo | n=209 | 63 (30–87) | 44.9% |  |  |  |  | 6.5months |
| Bruix | phase III | HCC | previous sorafenib | 567 | regorafenib 160 mg qd | n=374 | 64 (54–71) | 88.0% | NCT01774344 | 7 | multicentre | Bayer | 3.6months |
| 2017 |  |  |  |  | placebo | n=193 | 62 (55–68) | 88.0% |  |  |  |  | 1.9months |
| Bruix | phase III | HCC | resection or ablation | 1107 | sorafenib 400mg bid | n=559 | 58 (24–85) | 81.0% | NCT00692770 | 6 | multicentre | Bayer | 12.5months |
| 2015 |  |  |  |  | placebo | n=548 | 60 (19–83) | 83.0% |  |  |  |  | 22.2months |
| Cheng | phase III | HCC | previous systemic therapy | 224 | sorafenib 400mg bid | n=149 | 51 (23−86) | 84·7% | NCT00492752 | 6 | Asian | Bayer | NR |
| 2009 |  |  |  |  | placebo | n=75 | 52 (25−79) | 86·8% |  |  | multicentre |  | NR |
| Cheng | phase III | HCC | previous systemic therapy | 1068 | sunitinib 37.5mg qd | n=526 | 59(18-85） | 82.3% | NCT00699374 | 4 | multicentre | Bayer | NR |
| 2013 |  |  |  |  | sorafenib 400mg bid | n=542 | 59(18-84） | 84.4% |  |  |  |  | NR |
| Choueiri | phase II | RCC | not have received | 150 | cabozantinib 60mg qd | n=78 | 63(40-82) | 84.0% | NCT01835158 | 4 | multicentre | Pfizer | NR |
| 2017 |  |  | prior systemic treatment |  | sunitinib 50mg qd | n=72 | 64(31-87) | 73.0% |  |  |  |  | NR |
| Demetri | phase III | GIST | after failure of imatinib | 304 | sunitinib 50mg qd | n=202 | 58(23–84) | 63·8% | NCT00075218. | 6 | multicentre | Pfizer | NR |
| 2006 |  |  |  |  | placebo | n=102 | 55(23–81) | 61·0% |  |  |  |  | NR |
| Demetri | phase III | GIST | after failure of imatinib | 198 | regorafenib 160mg qd | n=132 | 60 (51–67) | 64.0% | NCT01271712 | 6 | multicentre | Bayer | 22.9weeks |
| 2013 |  |  | and sunitinib |  | placebo | n=66 | 61 (48–66) | 64.0% |  |  |  |  | 7.0weeks |
| Du Bois | phase III | OC | surgery, chemotherapy | 938 | pazopanib 800mg qd | n=477 | 56(25-85） | 0.0% | NCT00866697 | 4 | multicentre | NR | 8.9months |
| 2014 |  |  |  |  | placebo | n=461 | 57(20-85） | 0.0% |  |  |  |  | 11.7months |
| Eisen | phase II | RCC | previously untreated | 96 | nintedanib 200mg bid | n=64 | 62 (42–86) | 68.8% | NCT01024920 | 6 | multicentre | Pfizer, Bayer, | 348days |
| 2015 |  |  |  |  | sunitinib 50mg/kgqd | n=32 | 58 (29–79) | 68.8% |  |  |  | AstraZeneca | 391days |
| Elisei | phase III | TC | previous systemic regimens | 323 | cabozantinib 140mg qd | n =214 | 55(20-86） | 68.9% | NCT00704730 | 3 | multicentre | Bayer | 204days |
| 2013 |  |  |  |  | placebo | n =109 | 55(21-79） | 63.1% |  |  |  |  | 105days |
| Escudier | phase III | RCC | previous systemic regimens | 902 | sorafenib 400mg bid | n=451 | 58 (19–86) | 70.0% | NCT00073307 | 6 | multicentre | Bayer | 23week |
| 2007 |  |  |  |  | placebo | n=451 | 59 (29–84) | 75.0% |  |  |  |  | 12weeks |
| Grothey | phase III | CRC | previous systemic regimens | 753 | regorafenib 160mg qd | n=500 | 61 (54–67) | 62.0% | NCT01103323 | 6 | multicentre | Bayer | 2.8months |
| 2013 |  |  |  |  | placebo | n=253 | 61 (54–68) | 60.0% |  |  |  |  | 1.8months |
| Gross-Goupil | phase III | RCC | undergone nephrectomy | 715 | axitinib 5mg bid | n=356 | 58(51–66) | 77.0% | NCT01599754 | 4 | multicentre | Pfizer | NR |
| 2018 |  |  |  |  | placebo | n=359 | 58 (51–66) | 69.0% |  |  |  |  | NR |
| Haas | phase III | RCC | undergone nephrectomy | 1879 | sunitinib 50mg qd | n=625 | 56 (49–64) | 66.0% | NCT00326898 | 7 | multicentre | Pfizer, Bayer | 48weeks |
| 2016 |  |  |  |  | sorafenib 400mg bid | n=628 | 55 (48–63) | 67.0% |  |  |  |  | 48weeks |
|  |  |  |  |  | placebo | n=626 | 57 (49–64) | 68.0% |  |  |  |  | 54weeks |
| Herzog | phase II | OC | chemotherapy | 246 | sorafenib 400mg bid | n=123 | 56.9 | 0.0% | NCT00791778 | 4 | multicentre | Bayer | 17.6weeks |
| 2013 |  |  |  |  | placebo | n=123 | 54.4 | 0.0% |  |  |  |  | 51.9weeks |
| Hutson | phase III | RCC | undergone nephrectomy | 285 | axitinib 5mg bid | n=189 | 58 (23–83) | 70.0% | NCT00920816 | 4 | multicentre | Pfizer | 373days |
| 2013 |  |  |  |  | sorafenib 400mg bid | n=96 | 58(20–77) | 77.0% |  |  |  |  | 303.5dyas |
| Kang | phase II | HCC | previous systemic regimens | 201 | axitinib 5mg bid | n=133 | 61 (25–84) | 82.0% | NCT01210495 | 5 | multicentre | Pfizer | NR |
| 2015 |  |  |  |  | placebo | n=68 | 63 (26–83) | 82.0% |  |  |  |  | NR |
| Kudo | phase III | HCC | not received treatment | 951 | lenvatinib 12mg qd | n=476 | 63(20–88) | 85.0% | NCT01761266 | 4 | multicentre | Eisai | 5.7months |
| 2018 |  |  |  |  | sorafenib 400mg bid | n=475 | 62(22–88) | 84.0% |  |  |  |  | 3.7months |
| Kudo | phase III | HCC | after transarterial | 456 | sorafenib 400mg bid | n=229 | 69 | 76.0% | NCT00494299 | 3 | Japanese | Bayer | 17.1weeks |
| 2011 |  |  | chemoembolisation |  | placebo | n=227 | 70 | 73.4% |  |  | Korean |  | 20.1weeks |
| Leboulleux | phase II | TC | previous systemic regimens | 145 | vandetanib 300mg qd | n=73 | 63 (29–81) | 54.0% | NCT00537095 | 6 | multicentre | AstraZeneca | 192days |
| 2012 |  |  |  |  | placebo | n=72 | 64 (23–87) | 53.0% |  |  |  |  | 175.5days |
| Ledermann | phase II | OC | after chemotherapy | 83 | nintedanib 250mg bid | n =43 | 60(27-76) | 0.0% | NCT00710762 | 4 | multicentre | AstraZeneca | 2.8months |
| 2011 |  |  |  |  | placebo | n=40 | 63(40-75) | 0.0% |  |  |  |  | 2.8months |
| Lee | phase III | NSCLC | EGFR-TKI | 922 | vandetanib 300 mg qd | n=619 | 60(20-85) | 47.0% | NR | 4 | multicentre | AstraZeneca | 14.4weeks |
| 2012 |  |  |  |  | placebo | n=303 | 60(21-84) | 47.0% |  |  |  |  | 10.7weeks |
| Li | phase III | CRC | previous systemic regimens | 204 | regorafenib 160mg qd | n=136 | 57.5 | 63.0% | NCT01584830 | 6 | multicentre | Bayer | 4.0months |
| 2015 |  |  |  |  | placebo | n=68 | 55.5 | 49.0% |  |  |  |  | 1.6months |
| Llovet | phase III | HCC | previous systemic therapy | 499 | sorafenib 400mg bid | n=297 | 64.9 | 87.0% | NCT00105443 | 6 | multicentre | Bayer | 5.3months |
| 2008 |  |  |  |  | placebo | n=302 | 66.3 | 87.0% |  |  |  |  | 4.3months |
| Mir | phase II | STC | chemotherapy | 181 | regorafenib 160 mg qd | n=89 | * | * | NCT01900743 | 6 | Multicentre | Bayer | 1.6months |
| 2016 |  |  |  |  | placebo | n=92 | * | * |  |  |  |  | 1.9months |
| O’Brien | phase III | NSCLC | chemotherapy | 100 | pazopanib 800mg qd | n=50 | 64.6(25.9–80.7) | 48.1% | NCT01208064 | 4 | multicentre | NR | 14.07weeks |
| 2015 |  |  |  |  | placebo | n=50 | 64.2(28.4–81.1) | 42.0% |  |  |  |  | 14.43weeks |
| Palmer | phase II | HCC | previous systemic therapy | 93 | nintedanib 200mg bid | n=62 | 66 (34–86) | 77.4% | NCT01004003 | 4 | multicentre | Boehringer | 5.4months |
| 2018 |  |  |  |  | sorafenib 400mg bid | n=31 | 64 (28–83) | 83.9% |  |  |  |  | 5.42months |
| Pavlakis | phase II | GC | previous systemic therapy | 147 | regorafenib 160mg qd | n=97 | 63(33-81) | 80.0% | NR | 6 | multicentre | Bayer | 1.8months |
| 2016 |  |  |  |  | placebo | n=50 | 62(32-85) | 80.0% |  |  |  |  | 0.9months |
| Paz-Ares | phase III | NSCLC | chemotherapy | 697 | sorafenib 400mg bid | n=346 | 59 | 53.1% | NCT00863746 | 3 | multicentre | Bayer | 12weeks |
| 2015 |  |  |  |  | placebo | n=351 | 62 | 59.2% |  |  |  |  | 6.3weeks |
| Raymond | phase III | PNT | previous systemic therapy | 165 | sunitinib 37.5mg qd | n=83 | 56(25–84) | 49.0% | NCT00428597 | 4 | multicentre | Pfizer | 4.6months |
| 2011 |  |  |  |  | placebo | n=82 | 57(26–78) | 47.0% |  |  |  |  | 3.7months |
| Ravaud | phase III | RCC | undergone nephrectomy | 615 | sunitinib 50mg qd | n=309 | 57.0 (25–83) | 71.8% | NCT00375674 | 6 | multicentre | Pflizer | 12.39months |
| 2016 |  |  |  |  | placebo | n=306 | 58.0 (21–82) | 74.8% |  |  |  |  | 12.42months |
| Rini | phase III | RCC | chemotherapy | 714 | axitinib 5mg tid | n=359 | 61 (20–82) | 73.0% | NCT00678392 | 7 | multicentre | Pflizer | 6.4months |
| 2011 |  |  |  |  | sorafenib 400mg tid | n=355 | 61 (22–80) | 71.0% |  |  |  |  | 5.0months |
| Schlumberger | phase III | TC | prior systemic treatment | 392 | lenvatinib 24mg qd | n=261 | 64 | 47.9% | NCT01321554 | 6 | multicentre | Eisai | 17.1months |
| 2015 |  |  |  |  | placebo | n=131 | 61 | 57.3% |  |  |  |  | 17.4months |
| Sternberg | phase III | RCC | prior systemic treatment | 435 | pazopanib 800mg qd | n=290 | 59(28-85) | 68.0% | NCT00334282 | 4 | multicentre | GlaxoSmithKline | 7.6months |
| 2013 |  |  |  |  | placebo | n=145 | 60(25-81) | 75.0% |  |  |  |  | 3.8months |
| Sun | phase II | SCLC | chemotherapy | 95 | pazopanib 800mg qd | n=48 | 66.5 (57–79) | 83.3% | NCT0179784 | 6 | multicentre | Ministry of | NR |
| 2018 |  |  |  |  | placebo | n=47 | 67 (50–83) | 91.5% |  |  | Korea | Health & Welfare | NR |
| Van Cutsem | phase III | CRC | chemotherapy | 765 | nintedanib 200mg bid | n=384 | 62 (22–85) | 61.1% | NCT02149108 | 4 | multicentre | Boehringer | 2.1months |
| 2018 |  |  |  |  | placebo | n=381 | 62 (23–83) | 57.1% |  |  |  |  | 1.4months |
| Van Der Graaf | phase III | STC | chemotherapy | 362 | pazopanib 800mg qd | n=239 | 56.7 | 40.0% | NCT00753688 | 7 | multicentre | GlaxoSmithKline | 164weeks |
| 2012 |  |  |  |  | placebo | n=123 | 51.9 | 44.0% |  |  |  |  | 8.1weeks |
| Wells | phase III | TC | prior systemic therapy or not | 330 | vandetanib 300mg qd | n=231 | 50.7 | 58.0% | NCT00410761 | 4 | multicentre | AstraZeneca | 90.1weeks |
| 2012 |  |  |  |  | placebo | n=99 | 53.4 | 56.0% |  |  |  |  | 39.9weeks |
| Yen | phase II | HCC | no prior systemic therapy | 95 | nintedanib 200mg bid | n=63 | 58(33-84） | 90.5% | NCT01004003 | 5 | multicentre | Boehringer | 118days |
| 2018 |  |  |  |  | sorafenib 400mg bid | n=32 | 62(32-81) | 81.3% |  |  |  |  | 144days |
|  |  |  |  |  |  |  |  |  |  |  |  |  |  |

Abbreviation: HCC: hepatocellular carcinoma; NSCLC: non-small-cell lung cancer; SCLC: small cell lung cancer; RCC: renal cell carcinoma; GIST: gastrointestinal stromal tumor; OC: ovarian cancer; DT: desmoid tumors; CRC: colorectal cancer; TC: thyroid cancer; STC: soft tissue sarcoma; PNT: pancreatic neuroendocrine tumor; qd: once a day; bid: twice a day; tid: three times a day; NA: not available

**Reference**

1. Abou-Alfa GK, Meyer T, Cheng AL, et al. Cabozantinib in Patients with Advanced and Progressing Hepatocellular Carcinoma. New England journal of medicine 2018;379:54‐63.

2. Ahn JS, Lee KH, Sun JM, et al. A randomized, phase II study of vandetanib maintenance for advanced or metastatic non-small-cell lung cancer following first-line platinum-doublet chemotherapy. Lung cancer (Amsterdam, Netherlands) 2013;82:455-460.

3. Arnold AM, Seymour L, Smylie M, et al. Phase II study of vandetanib or placebo in small-cell lung cancer patients after complete or partial response to induction chemotherapy with or without radiation therapy: national Cancer Institute of Canada Clinical Trials Group Study BR.20. Journal of clinical oncology 2007;25:4278‐4284.

4. Baggstrom MQ, Socinski MA, Wang XF, et al. Maintenance Sunitinib following Initial Platinum-Based Combination Chemotherapy in Advanced-Stage IIIB/IV Non–Small Cell Lung Cancer: A Randomized, Double-Blind, Placebo-Controlled Phase III Study—CALGB 30607 (Alliance). Journal of Thoracic Oncology 2017;12:843-849.

5. Brose MS, Nutting CM, Jarzab B, et al. Sorafenib in radioactive iodine-refractory, locally advanced or metastatic differentiated thyroid cancer: a randomised, double-blind, phase 3 trial. Lancet (London, England) 2014;384:319‐328.

6. Bruix J, Qin S, Merle P, et al. Regorafenib for patients with hepatocellular carcinoma who progressed on sorafenib treatment (RESORCE): a randomised, double-blind, placebo-controlled, phase 3 trial. Lancet (London, England) 2017;389:56‐66.

7. Bruix J, Takayama T, Mazzaferro V, et al. Adjuvant sorafenib for hepatocellular carcinoma after resection or ablation (STORM): A phase 3, randomised, double-blind, placebo-controlled trial. The Lancet Oncology 2015;16:1344-1354.

8. Cheng AL, Kang YK, Chen Z, et al. Efficacy and safety of sorafenib in patients in the Asia-Pacific region with advanced hepatocellular carcinoma: a phase III randomised, double-blind, placebo-controlled trial. The Lancet Oncology 2009;10:25-34.

9. Cheng AL, Kang YK, Lin DY, et al. Sunitinib versus sorafenib in advanced hepatocellular cancer: results of a randomized phase III trial. Journal of clinical oncology 2013;31:4067‐4075.

10. Choueiri TK, Halabi S, Sanford BL, et al. Cabozantinib versus sunitinib as initial targeted therapy for patients with metastatic renal cell carcinoma of poor or intermediate risk: The alliance A031203 CABOSUN trial. Journal of Clinical Oncology 2017;35:591-597.

11. Demetri GD, van Oosterom AT, Garrett CR, et al. Efficacy and safety of sunitinib in patients with advanced gastrointestinal stromal tumour after failure of imatinib: a randomised controlled trial. Lancet (London, England) 2006;368:1329‐1338.

12. Demetri GD, Reichardt P, Kang YK, et al. Efficacy and safety of regorafenib for advanced gastrointestinal stromal tumours after failure of imatinib and sunitinib (GRID): an international, multicentre, randomised, placebo-controlled, phase 3 trial. Lancet (London, England) 2013;381:295‐302.

13. Du Bois A, Floquet A, Kim JW, et al. Incorporation of pazopanib in maintenance therapy of ovarian cancer. Journal of Clinical Oncology 2014;32:3374-3381.

14. Eisen T, Loembé AB, Shparyk Y, et al. A randomised, phase II study of nintedanib or sunitinib in previously untreated patients with advanced renal cell cancer: 3-year results. British journal of cancer 2015;113:1140‐1147.

15. Elisei R, Schlumberger MJ, Müller SP, et al. Cabozantinib in progressive medullary thyroid cancer. Journal of Clinical Oncology 2013;31:3639-3646.

16. Escudier B, Eisen T, Stadler WM, et al. Sorafenib in advanced clear-cell renal-cell carcinoma. New England journal of medicine 2007;356:125‐134.

17. Gounder MM, Mahoney MR, Van Tine BA, et al. Sorafenib for Advanced and Refractory Desmoid Tumors. New England journal of medicine 2018;379:2417‐2428.

18. Grothey A, Van Cutsem E, Sobrero A, et al. Regorafenib monotherapy for previously treated metastatic colorectal cancer (CORRECT): An international, multicentre, randomised, placebo-controlled, phase 3 trial. The Lancet 2013;381:303-312.

19. Gross-Goupil M, Kwon TG, Eto M, et al. Axitinib vs placebo in patients at high risk of recurrent renal cell carcinoma (RCC): ATLAS trial results. Annals of Oncology 2018;29:viii303.

20. Haas NB, Manola J, Uzzo RG, et al. Adjuvant sunitinib or sorafenib for high-risk, non-metastatic renal-cell carcinoma (ECOG-ACRIN E2805): A double-blind, placebo-controlled, randomised, phase 3 trial. The Lancet 2016;387:2008-2016.

21. Herzog TJ, Scambia G, Kim BG, et al. A randomized phase II trial of maintenance therapy with Sorafenib in front-line ovarian carcinoma. Gynecologic oncology 2013;130:25‐30.

22. Hutson TE, Lesovoy V, Al-Shukri S, et al. Axitinib versus sorafenib as first-line therapy in patients with metastatic renal-cell carcinoma: a randomised open-label phase 3 trial. The lancet Oncology 2013;14:1287‐1294.

23. Kang YK, Yau T, Park JW, et al. Randomized phase II study of axitinib versus placebo plus best supportive care in second-line treatment of advanced hepatocellular carcinoma. Annals of oncology : official journal of the european society for medical oncology 2015;26:2457‐2463.

24. Kudo M, Finn RS, Qin S, et al. Lenvatinib versus sorafenib in first-line treatment of patients with unresectable hepatocellular carcinoma: a randomised phase 3 non-inferiority trial. Lancet (London, England) 2018;391:1163‐1173.

25. Kudo M, Imanaka K, Chida N, et al. Phase III study of sorafenib after transarterial chemoembolisation in Japanese and Korean patients with unresectable hepatocellular carcinoma. European journal of cancer (Oxford, England : 1990) 2011;47:2117‐2127.

26. Leboulleux S, Bastholt L, Krause T, et al. Vandetanib in locally advanced or metastatic differentiated thyroid cancer: A randomised, double-blind, phase 2 trial. The Lancet Oncology 2012;13:897-905.

27. Ledermann JA, Hackshaw A, Kaye S, et al. Randomized phase II placebo-controlled trial of maintenance therapy using the oral triple angiokinase inhibitor BIBF 1120 after chemotherapy for relapsed ovarian cancer. Journal of clinical oncology : official journal of the American Society of Clinical Oncology 2011;29:3798-3804.

28. Lee JS, Hirsh V, Park K, et al. Vandetanib Versus placebo in patients with advanced non-small-cell lung cancer after prior therapy with an epidermal growth factor receptor tyrosine kinase inhibitor: a randomized, double-blind phase III trial (ZEPHYR). Journal of clinical oncology 2012;30:1114‐1121.

29. Li J, Qin S, Xu R, et al. Regorafenib plus best supportive care versus placebo plus best supportive care in Asian patients with previously treated metastatic colorectal cancer (CONCUR): a randomised, double-blind, placebo-controlled, phase 3 trial. The lancet Oncology 2015;16:619‐629.

30. Llovet JM, Ricci S, Mazzaferro V, et al. Sorafenib in advanced hepatocellular carcinoma. New England journal of medicine 2008;359:378‐390.

31. Mir O, Brodowicz T, Italiano A, et al. Safety and efficacy of regorafenib in patients with advanced soft tissue sarcoma (REGOSARC): a randomised, double-blind, placebo-controlled, phase 2 trial. The lancet Oncology 2016;17:1732‐1742.

32. O'Brien MER, Gaafar R, Hasan B, et al. Maintenance pazopanib versus placebo in Non-Small Cell Lung Cancer patients non-progressive after first line chemotherapy: A double blind randomised phase III study of the lung cancer group, EORTC 08092 (EudraCT: 2010-018566-23, NCT01208064). European Journal of Cancer 2015;51:1511-1528.

33. Palmer DH, Ma YT, Peck-Radosavljevic M, et al. A multicentre, open-label, phase-I/randomised phase-II study to evaluate safety, pharmacokinetics, and efficacy of nintedanib vs. sorafenib in European patients with advanced hepatocellular carcinoma. British journal of cancer 2018;118:1162-1168.

34. Pavlakis N, Sjoquist KM, Martin AJ, et al. Regorafenib for the Treatment of Advanced Gastric Cancer (INTEGRATE): a Multinational Placebo-Controlled Phase II Trial. Journal of clinical oncology 2016;34:2728‐2735.

35. Paz-Ares L, Hirsh V, Zhang L, et al. Monotherapy Administration of Sorafenib in Patients with Non-Small Cell Lung Cancer (MISSION) Trial: A Phase III, Multicenter, Placebo-Controlled Trial of Sorafenib in Patients with Relapsed or Refractory Predominantly Nonsquamous Non-Small-Cell Lung Cancer after 2 or 3 Previous Treatment Regimens. Journal of Thoracic Oncology 2015;10:1745-1753.

36. Raymond E, Dahan L, Raoul JL, et al. Sunitinib malate for the treatment of pancreatic neuroendocrine tumors. New England journal of medicine 2011;364:501‐513.

37. Ravaud A, Motzer RJ, Pandha HS, et al. Adjuvant sunitinib in high-risk renal-cell carcinoma after nephrectomy. New England Journal of Medicine 2016;375:2246-2254.

38. Rini BI, Escudier B, Tomczak P, et al. Comparative effectiveness of axitinib versus sorafenib in advanced renal cell carcinoma (AXIS): A randomised phase 3 trial. The Lancet 2011;378:1931-1939.

39. Schlumberger M, Tahara M, Wirth LJ, et al. Lenvatinib versus placebo in radioiodine-refractory thyroid cancer. New England Journal of Medicine 2015;372:621-630.

40. Sternberg CN, Hawkins RE, Wagstaff J, et al. A randomised, double-blind phase III study of pazopanib in patients with advanced and/or metastatic renal cell carcinoma: final overall survival results and safety update. European journal of cancer (Oxford, England : 1990) 2013;49:1287‐1296.

41. Sun JM, Lee KH, Kim BS, et al. Pazopanib maintenance after first-line etoposide and platinum chemotherapy in patients with extensive disease small-cell lung cancer: a multicentre, randomised, placebo-controlled Phase II study (KCSG-LU12-07). British journal of cancer 2018;118:648‐653.

42. Van Cutsem E, Yoshino T, Lenz HJ, et al. Nintedanib for the treatment of patients with refractory metastatic colorectal cancer (LUME-Colon 1): a phase III, international, randomized, placebo-controlled study. Annals of oncology : official journal of the european society for medical oncology 2018;29:1955‐1963.

43. Van Der Graaf WTA, Blay JY, Chawla SP, et al. Pazopanib for metastatic soft-tissue sarcoma (PALETTE): A randomised, double-blind, placebo-controlled phase 3 trial. The Lancet 2012;379:1879-1886.

44. Wells Jr SA, Robinson BG, Gagel RF, et al. Vandetanib in patients with locally advanced or metastatic medullary thyroid cancer: A randomized, double-blind phase III trial. Journal of Clinical Oncology 2012;30:134-141.

45. Yen CJ, Kim TY, Feng YH, et al. A Phase I/Randomized Phase II Study to Evaluate the Safety, Pharmacokinetics, and Efficacy of Nintedanib versus Sorafenib in Asian Patients with Advanced Hepatocellular Carcinoma. Liver Cancer 2018;7:165-178.
