# Supplementary material for: Comparative evaluation of cardiovascular risks among nine FDA-approved VEGFR-TKIs in patients with solid tumors: a Bayesian network analysis of randomized controlled trials
Source: J Cancer Res Clin Oncol. 2021 Mar 16;147(8):2407–20. doi: 10.1007/s00432-021-03521-w (PMC8236482; doi:10.1007/s00432-021-03521-w)
Supplement: Supplementary file 3 — Supplementary file3 (DOCX 19 KB) [file 432_2021_3521_MOESM3_ESM.docx]

| **Study ID** | **Comorbid cardiac disease** | **Cardiac related risk** | **Adverse events defining criterion** |
| --- | --- | --- | --- |
| Abou-Alfa 2018 | low risk | unknown | CTCAE v4.0 |
| Ahn 2013 | unknown | low risk | CTCAE v3.0 |
| Arnold 2017 | low risk | unknown | unkonwn |
| Baggstrom 2017 | low risk | low risk | CTCAE v3.0 |
| Brose 2014 | unknown | unknown | CTCAE v3.0 |
| Bruix 2017 | low risk | unknown | CTCAE v4.03 |
| Bruix 2015 | low risk | unknown | unkonwn |
| Cheng 2009 | low risk | unknown | CTCAE v3.0 |
| Cheng 2013 | low risk | unknown | CTCAE v3.0 |
| Choueiri 2017 | unknown | unknown | CTCAE v4.0 |
| Demetri 2006 | low risk | unknown | CTCAE v3.0 |
| Demetri 2013 | low risk | unknown | CTCAE v4.0 |
| Du Bois 2014 | low risk | unknown | CTCAE v4.0 |
| Eisen 2015 | unknown | unknown | CTCAE v3.0 |
| Elisei 2013 | low risk | unknown | CTCAE v3.0 |
| Escudier 2007 | unknown | unknown | CTCAE v3.0 |
| Gounder 2018 | unknown | unknown | CTCAE v4.03 |
| Grothey 2013 | low risk | unknown | CTCAE v3.0 |
| Gross-Goupil 2018 | unknown | unknown | CTCAE v4.0 |
| Haas 2016 | low risk | unknown | CTCAE v3.0 |
| Herzog 2013 | unknown | unknown | CTCAE v3.0 |
| Hutson 2013 | low risk | unknown | CTCAE v3.0 |
| Kang 2015 | unknown | unknown | CTCAE v3.0 |
| Kudo 2018 | low risk | unknown | CTCAE v4.0 |
| Kudo 2011 | low risk | unknown | CTCAE v3.0 |
| Leboulleux 2012 | low risk | unknown | CTCAE v3.0 |
| Ledermann 2011 | low risk | unknown | CTCAE v3.0 |
| Lee 2012 | low risk | low risk | CTCAE v3.0 |
| Li 2015 | low risk | unknown | CTCAE v4.0 |
| Llovet 2008 | unknown | unknown | CTCAE v3.0 |
| Mir 2016 | low risk | low risk | CTCAE v4.03 |
| O’Brien 2015 | unknown | low risk | unknown |
| Palmer 2018 | unknown | unknown | CTCAE v3.0 |
| Pavlakis 2016 | low risk | unknown | CTCAE v4.0 |
| Paz-Ares 2015 | unknown | low risk | CTCAE v3.0 |
| Raymond 2011 | low risk | unknown | CTCAE v3.0 |
| Ravaud 2016 | low risk | unknown | CTCAE v3.0 |
| Rini 2011 | low risk | unknown | CTCAE v3.0 |
| Schlumberger 2015 | low risk | unknown | CTCAE v4.0 |
| Sternberg 2013 | low risk | unknown | CTCAE v3.0 |
| Sun 2018 | unknown | low risk | CTCAE v4.0 |
| Van Cutsem 2018 | low risk | unknown | CTCAE v3.0 |
| Van Der Graaf 2012 | low risk | unknown | CTCAE v3.0 |
| Wells 2012 | low risk | unknown | CTCAE v3.0 |
| Yen 2018 | unknown | unknown | CTCAE v3.0 |
|  |  |  |  |
