# Supplementary material for: Comparative evaluation of cardiovascular risks among nine FDA-approved VEGFR-TKIs in patients with solid tumors: a Bayesian network analysis of randomized controlled trials
Source: J Cancer Res Clin Oncol. 2021 Mar 16;147(8):2407–20. doi: 10.1007/s00432-021-03521-w (PMC8236482; doi:10.1007/s00432-021-03521-w)
Supplement: Supplementary file 4 — Supplementary file4 (DOCX 34 KB) [file 432_2021_3521_MOESM4_ESM.docx]

**Supplement Table 1** PRISMA NMA checklist

**Supplement Table 2** Characteristics of involved trials

**Supplement Table 3** Cardiovascular related characteristic risk evaluation of involved trials

**Supplement Table 4** The PSRF in network of cardiovascular event (all grades)

**Supplement Table 5** The node-splitting analysis of all grades cardiovascular event’s direct comparison and indirect comparison groups

**Supplement Table 6** The PSRF in network of serious cardiovascular event (grade 3 or higher)

**Supplement Table 7** The node-splitting analysis of serious cardiovascular event’s direct comparison and indirect comparison groups

**Supplement Table 8** The PSRF in network of hypertension (all grades)

**Supplement Table 9** The node-splitting analysis of all hypertension event’s direct comparison and indirect comparison groups

**Supplement Table 10** The PSRF in network of serious hypertension (grade 3 or higher)

**Supplement Table 11** The node-splitting analysis of serious hypertension event’s direct comparison and indirect comparison groups

**Supplement Table 12** The PSRF in network of cardiac event

**Supplement Table 13** The node-splitting analysis of cardiac event’s direct comparison and indirect comparison groups

**Supplement Fig 1** The forest plot of the pairwise meta-analysis of VEGFR-TKIs’ cardiovascular event (all grades). OR= odds ratios CI= confidence interval

**Supplement Fig 2** The probability of all grades cardiovascular event of nine VEGFR-TKIs

**Supplement Fig 3** The forest plot of the pairwise meta-analysis of VEGFR-TKIs’ serious cardiovascular event. OR= odds ratios CI= confidence interval

**Supplement Fig 4** The probability of serious cardiovascular event (grade 3 or higher) in different VEGFR-TKIs

**Supplement Fig 5** The forest plot of the pairwise meta-analysis of VEGFR-TKIs’ hypertension (all grades). OR= odds ratios CI= confidence interval

**Supplement Fig 6** The probability of hypertension (all grades) of nine VEGFR-TKIs

**Supplement Fig 7** The forest plot of the pairwise meta-analysis of VEGFR-TKIs’ serious hypertension. OR= odds ratios CI= confidence interval

**Supplement Fig 8** The probability of serious hypertension (grade 3 or higher) of nine VEGFR-TKIs

**Supplement Fig 9** The forest plot of the pairwise meta-analysis of VEGFR-TKIs’ cardiac event. OR= odds ratios CI= confidence interval

**Supplement Fig 10** The probability of cardiac event of nine VEGFR-TKIs

| **Parameter** | **PSRF** |
| --- | --- |
| d.PLA.AXI | 1.00 |
| d.PLA.CAB | 1.00 |
| d.PLA.LEN | 1.00 |
| d.PLA.NIN | 1.00 |
| d.PLA.PAZ | 1.00 |
| d.PLA.REG | 1.00 |
| d.PLA.SOR | 1.00 |
| d.PLA.SUN | 1.00 |
| d.PLA.VAN | 1.00 |
| sd.d | 1.01 |

**Supplement Table 4** The PSRF in network of cardiovascular event (all grades)

| **Name** | **Direct Effect** | **Indirect Effect** | **Overall** | **P-Value** |
| --- | --- | --- | --- | --- |
| AXI, PLA | -1.87 (-2.55, -1.23) | -2.31 (-2.99, -1.67) | -2.09 (-2.57, -1.66) | 0.33 |
| AXI, SOR | -0.65 (-1.26, -0.05) | -0.22 (-0.91, 0.47) | -0.47 (-0.92, -0.02) | 0.33 |
| CAB, PLA | -2.04 (-2.76, -1.35) | -2.23 (-3.38, -1.10) | -2.10 (-2.68, -1.52) | 0.78 |
| CAB, SUN | -0.70 (-1.77, 0.35) | -0.51 (-1.34, 0.30) | -0.57 (-1.20, 0.06) | 0.79 |
| LEN, PLA | -3.17 (-4.10, -2.22) | -2.45 (-3.29, -1.68) | -2.75 (-3.40, -2.14) | 0.26 |
| LEN, SOR | -0.88 (-1.66, -0.10) | -1.59 (-2.57, -0.58) | -1.13 (-1.75, -0.53) | 0.24 |
| NIN, PLA | -0.44 (-1.20, 0.27) | -1.16 (-1.89, -0.44) | -0.80 (-1.35, -0.27) | 0.17 |
| NIN, SOR | 0.48 (-0.41, 1.38) | 1.00 (0.31, 1.65) | 0.82 (0.29, 1.36) | 0.35 |
| NIN, SUN | 0.41 (-0.73, 1.55) | 0.85 (0.11, 1.50) | 0.73 (0.11, 1.31) | 0.51 |
| PLA, SOR | 1.72 (1.41, 2.05) | 1.37 (0.89, 1.87) | 1.62 (1.37, 1.90) | 0.22 |
| PLA, SUN | 1.45 (0.98, 1.91) | 1.60 (0.96, 2.25) | 1.53 (1.16, 1.89) | 0.69 |
| SOR, SUN | 0.14 (-0.43, 0.72) | -0.24 (-0.78, 0.29) | -0.09 (-0.50, 0.27) | 0.33 |

**Supplement Table 5** The node-splitting analysis of all grades cardiovascular event’s direct comparison and indirect comparison groups

| **Parameter** | **PSRF** |
| --- | --- |
| d.PLA.CAB | 1.00 |
| d.PLA.LEN | 1.00 |
| d.PLA.NIN | 1.00 |
| d.PLA.PAZ | 1.00 |
| d.PLA.REG | 1.00 |
| d.PLA.SOR | 1.00 |
| d.PLA.SUN | 1.00 |
| d.PLA.VAN | 1.00 |
| d.PLA.AXI | 1.00 |
| sd.d | 1.01 |

**Supplement Table 6** The PSRF in network of serious cardiovascular event (grade 3 or higher)

| **Name** | **Direct Effect** | **Indirect Effect** | **Overall** | **P-Value** |
| --- | --- | --- | --- | --- |
| CAB, PLA | -2.55 (-4.12, -1.23) | -2.24 (-4.13, -0.49) | -2.43 (-3.52, -1.42) | 0.76 |
| CAB, SUN | -0.33 (-1.89, 1.24) | -0.58 (-2.25, 1.01) | -0.47 (-1.55, 0.66) | 0.8 |
| LEN, SOR | -0.96 (-1.96, 0.05) | -3.55 (-5.05, -2.07) | -1.85 (-3.02, -0.82) | 0.01 |
| LEN, SUN | -3.15 (-4.51, -1.93) | -0.59 (-1.81, 0.60) | -1.89 (-3.07, -0.79) | 0.01 |
| NIN, PLA | -1.91 (-3.49, -0.44) | -1.91 (-3.51, -0.47) | -1.90 (-3.03, -0.88) | 1 |
| NIN, SOR | -0.35 (-2.42, 1.48) | 0.30 (-1.09, 1.66) | 0.09 (-1.01, 1.16) | 0.58 |
| NIN, SUN | 0.51 (-1.50, 2.55) | -0.18 (-1.59, 1.21) | 0.07 (-1.10, 1.19) | 0.57 |
| PLA, SOR | 1.90 (1.26, 2.60) | 2.38 (1.04, 3.69) | 2.00 (1.40, 2.64) | 0.53 |
| PLA, SUN | 2.18 (1.13, 3.40) | 1.79 (0.78, 2.86) | 1.97 (1.26, 2.76) | 0.61 |
| SOR, SUN | 0.37 (-1.19, 1.87) | -0.21 (-1.15, 0.79) | -0.03 (-0.82, 0.77) | 0.52 |

**Supplement Table 7** The node-splitting analysis of serious cardiovascular event’s direct comparison and indirect comparison groups

| **Parameter** | **PSRF** |
| --- | --- |
| d.PLA.AXI | 1.00 |
| d.PLA.CAB | 1.00 |
| d.PLA.LEN | 1.00 |
| d.PLA.NIN | 1.00 |
| d.PLA.PAZ | 1.00 |
| d.PLA.REG | 1.00 |
| d.PLA.SOR | 1.00 |
| d.PLA.SUN | 1.00 |
| d.PLA.VAN | 1.00 |
| sd.d | 1.00 |

**Supplement Table 8** The PSRF in network of hypertension (all grades)

| **Name** | **Direct Effect** | **Indirect Effect** | **Overall** | **P-Value** |
| --- | --- | --- | --- | --- |
| AXI, PLA | -1.83 (-2.44, -1.28) | -2.31 (-2.95, -1.75) | -2.06 (-2.50, -1.65) | 0.23 |
| AXI, SOR | -0.64 (-1.20, -0.12) | -0.14 (-0.80, 0.50) | -0.43 (-0.86, -0.01) | 0.23 |
| CAB, PLA | -2.09 (-2.82, -1.41) | -2.36 (-3.41, -1.26) | -2.15 (-2.75, -1.58) | 0.69 |
| CAB, SUN | -0.71 (-1.70, 0.33) | -0.43 (-1.24, 0.31) | -0.53 (-1.17, 0.09) | 0.67 |
| LEN, PLA | -3.10 (-4.07, -2.19) | -2.48 (-3.28, -1.72) | -2.70 (-3.32, -2.16) | 0.29 |
| LEN, SOR | -0.87 (-1.59, -0.17) | -1.47 (-2.43, -0.51) | -1.07 (-1.67, -0.52) | 0.3 |
| NIN, PLA | -0.55 (-1.42, 0.21) | -1.53 (-2.32, -0.69) | -1.05 (-1.68, -0.48) | 0.12 |
| NIN, SOR | 0.03 (-0.94, 0.96) | 0.94 (0.13, 1.69) | 0.59 (-0.04, 1.17) | 0.14 |
| NIN, SUN | 0.41 (-1.13, 1.82) | 0.62 (-0.17, 1.32) | 0.58 (-0.11, 1.20) | 0.79 |
| PLA, SOR | 1.75 (1.46, 2.07) | 1.36 (0.88, 1.82) | 1.63 (1.40, 1.90) | 0.14 |
| PLA, SUN | 1.57 (1.14, 2.01) | 1.71 (1.08, 2.32) | 1.62 (1.28, 1.96) | 0.7 |
| SOR, SUN | 0.12 (-0.41, 0.64) | -0.15 (-0.68, 0.37) | -0.01 (-0.39, 0.34) | 0.46 |

**Supplement Table 9** The node-splitting analysis of all hypertension event’s direct comparison and indirect comparison groups

| Parameter | PSRF |
| --- | --- |
| d.PLA.AXI | 1.00 |
| d.PLA.CAB | 1.00 |
| d.PLA.LEN | 1.00 |
| d.PLA.NIN | 1.00 |
| d.PLA.PAZ | 1.00 |
| d.PLA.REG | 1.00 |
| d.PLA.SOR | 1.00 |
| d.PLA.SUN | 1.00 |
| d.PLA.VAN | 1.01 |
| sd.d | 1.00 |

**Supplement Table 10** The PSRF in network of serious hypertension (grade 3 or higher)

| **Name** | **Direct Effect** | **Indirect Effect** | **Overall** | **P-Value** |
| --- | --- | --- | --- | --- |
| AXI, PLA | -3.31 (-5.93, -1.30) | -2.70 (-3.98, -1.73) | -2.81 (-3.97, -1.93) | 0.62 |
| AXI, SOR | -0.97 (-2.11, -0.12) | -1.83 (-5.28, 0.45) | -1.01 (-2.06, -0.25) | 0.54 |
| CAB, PLA | -2.95 (-4.61, -1.67) | -2.82 (-4.46, -1.29) | -2.91 (-4.04, -1.91) | 0.88 |
| CAB, SUN | -0.32 (-1.68, 0.98) | -0.43 (-2.13, 1.29) | -0.38 (-1.41, 0.69) | 0.92 |
| LEN, PLA | -3.51 (-5.23, -1.98) | -2.69 (-4.03, -1.52) | -2.97 (-4.00, -2.07) | 0.38 |
| LEN, SOR | -0.93 (-2.05, 0.12) | -1.87 (-3.57, -0.23) | -1.17 (-2.14, -0.29) | 0.34 |
| NIN, PLA | -1.94 (-3.86, -0.65) | -2.26 (-3.92, -0.56) | -2.06 (-3.34, -1.01) | 0.85 |
| NIN, SOR | -1.39 (-4.73, 0.90) | 0.01 (-1.47, 1.44) | -0.27 (-1.54, 0.82) | 0.32 |
| NIN, SUN | 1.52 (-1.29, 5.36) | 0.08 (-1.55, 1.55) | 0.47 (-0.87, 1.70) | 0.39 |
| PLA, SOR | 1.60 (1.07, 2.26) | 2.26 (1.27, 3.26) | 1.79 (1.31, 2.32) | 0.27 |
| PLA, SUN | 2.70 (1.58, 4.10) | 2.46 (1.42, 3.62) | 2.54 (1.79, 3.39) | 0.75 |
| SOR, SUN | 0.32 (-0.99, 1.64) | 1.06 (0.02, 2.22) | 0.75 (-0.05, 1.60) | 0.33 |

**Supplement Table 11** The node-splitting analysis of serious hypertension event’s direct comparison and indirect comparison groups

| Parameter | PSRF |
| --- | --- |
| d.PLA.AXI | 1.00 |
| d.PLA.NIN | 1.00 |
| d.PLA.PAZ | 1.00 |
| d.PLA.REG | 1.00 |
| d.PLA.SOR | 1.00 |
| d.PLA.SUN | 1.00 |
| d.PLA.VAN | 1.01 |
| sd.d | 1.02 |

**Supplement Table 12** The PSRF in network of cardiac event

| **Name** | **Direct Effect** | **Indirect Effect** | **Overall** | **P-Value** |
| --- | --- | --- | --- | --- |
| AXI, PLA | -0.87 (-4.63, 2.20) | -1.38 (-5.14, 1.44) | -1.02 (-3.43, 0.91) | 0.82 |
| AXI, SOR | -0.71 (-4.24, 2.06) | -0.22 (-4.16, 3.02) | -0.32 (-2.60, 1.68) | 0.82 |
| NIN, PLA | 0.28 (-1.74, 2.24) | -0.02 (-1.91, 2.03) | 0.19 (-1.01, 1.36) | 0.78 |
| NIN, SOR | 1.88 (-1.26, 6.09) | 0.75 (-0.79, 2.38) | 0.85 (-0.29, 2.32) | 0.54 |
| NIN, SUN | 0.45 (-1.61, 2.43) | 1.23 (-0.45, 3.57) | 0.85 (-0.33, 2.25) | 0.50 |
| PLA, SOR | 0.85 (-0.13, 2.11) | 0.36 (-1.23, 2.44) | 0.68 (-0.04, 1.74) | 0.60 |
| PLA, SUN | 0.63 (-0.81, 2.90) | 0.78 (-0.94, 2.60) | 0.66 (-0.29, 1.90) | 0.89 |
| SOR, SUN | 0.40 (-1.37, 2.32) | -0.44 (-1.99, 1.17) | -0.00 (-1.20, 1.09) | 0.40 |

**Supplement Table 13** The node-splitting analysis of cardiac event’s direct comparison and indirect comparison groups
