# Supplementary figures and images for: Comparative evaluation of cardiovascular risks among nine FDA-approved VEGFR-TKIs in patients with solid tumors: a Bayesian network analysis of randomized controlled trials
Source: J Cancer Res Clin Oncol. 2021 Mar 16;147(8):2407–20. doi: 10.1007/s00432-021-03521-w (PMC8236482; doi:10.1007/s00432-021-03521-w)

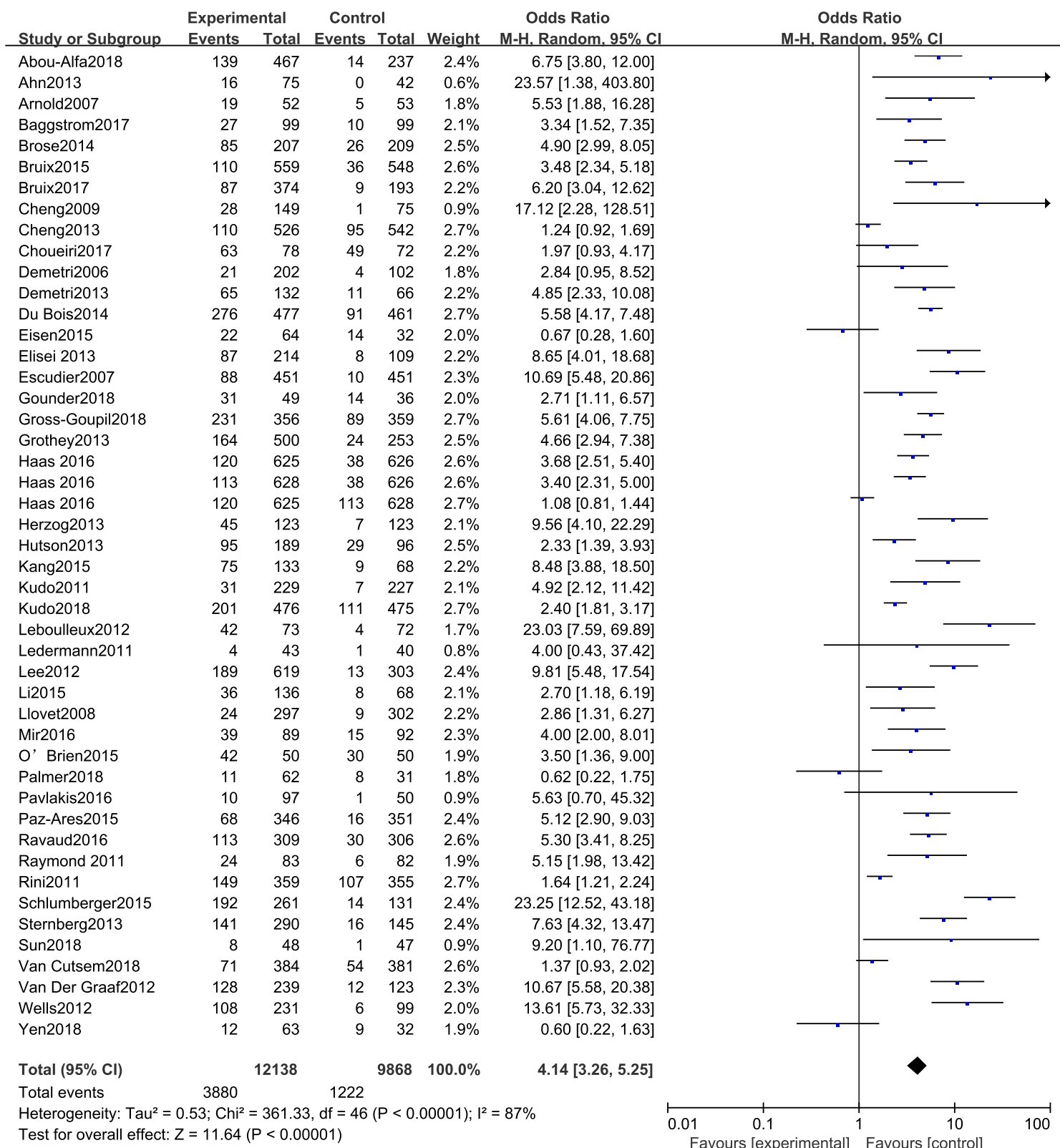

Supplement: Supplementary file 5 — Supplementary file5 (PDF 1537 KB) [file 432_2021_3521_MOESM5_ESM.pdf]

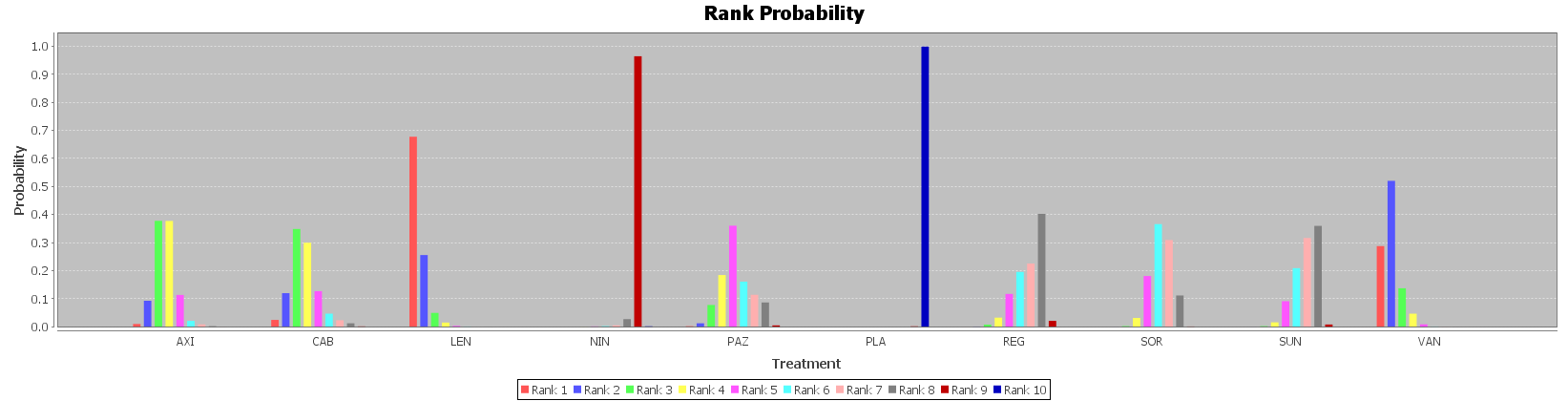

Supplement: Supplementary file 6 — Supplementary file6 (PNG 20 KB) [file 432_2021_3521_MOESM6_ESM.png]

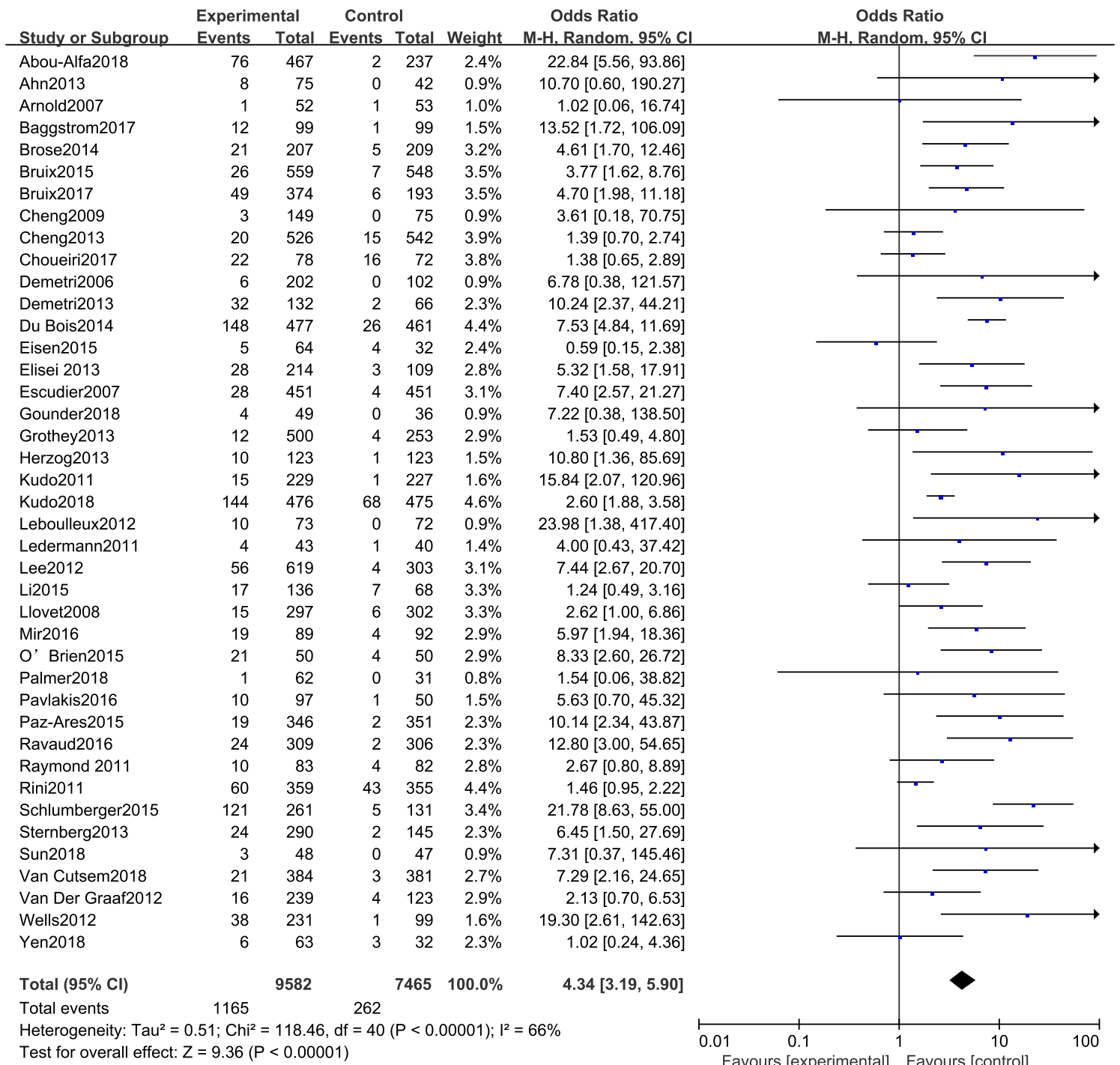

Supplement: Supplementary file 7 — Supplementary file7 (PDF 1330 KB) [file 432_2021_3521_MOESM7_ESM.pdf]

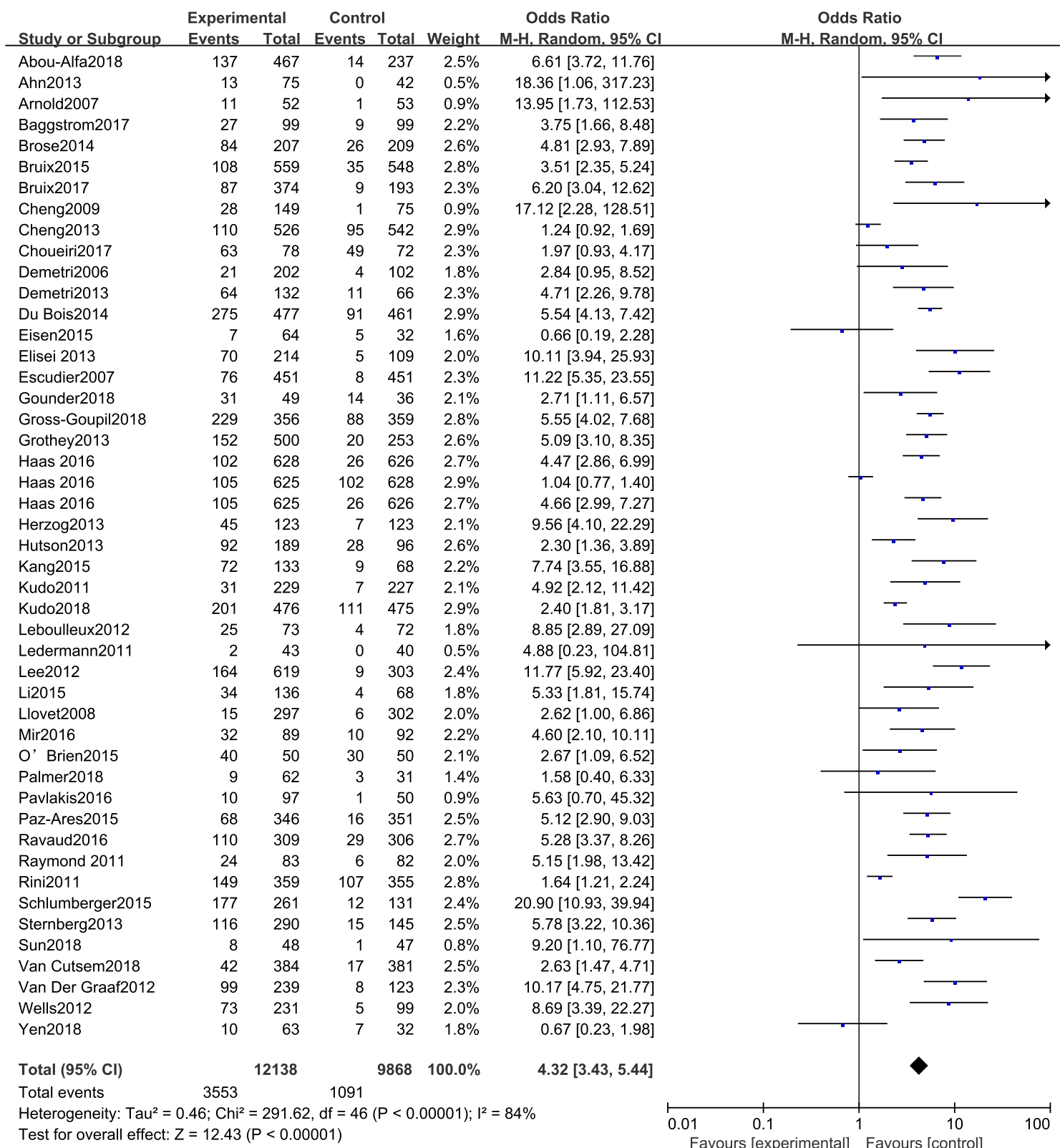

Supplement: Supplementary file 8 — Supplementary file8 (PDF 1522 KB) [file 432_2021_3521_MOESM8_ESM.pdf]

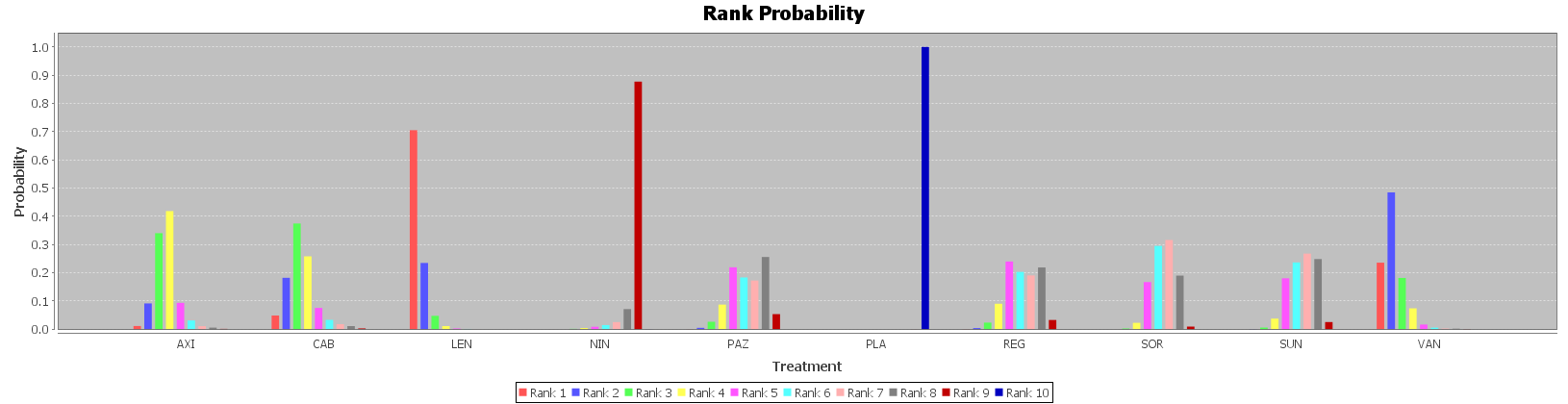

Supplement: Supplementary file 9 — Supplementary file9 (PNG 20 KB) [file 432_2021_3521_MOESM9_ESM.png]

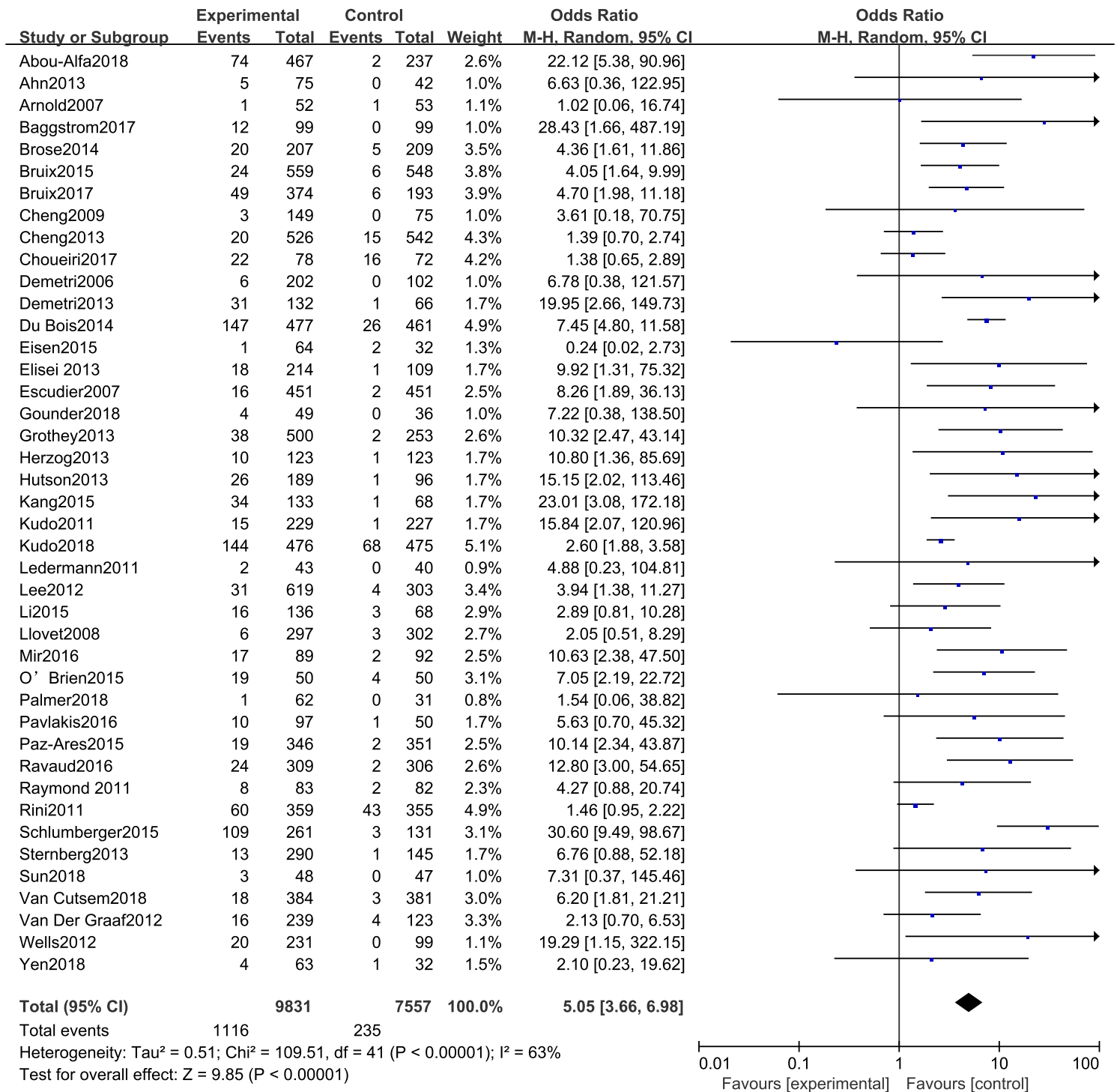

Supplement: Supplementary file 10 — Supplementary file10 (PDF 1362 KB) [file 432_2021_3521_MOESM10_ESM.pdf]

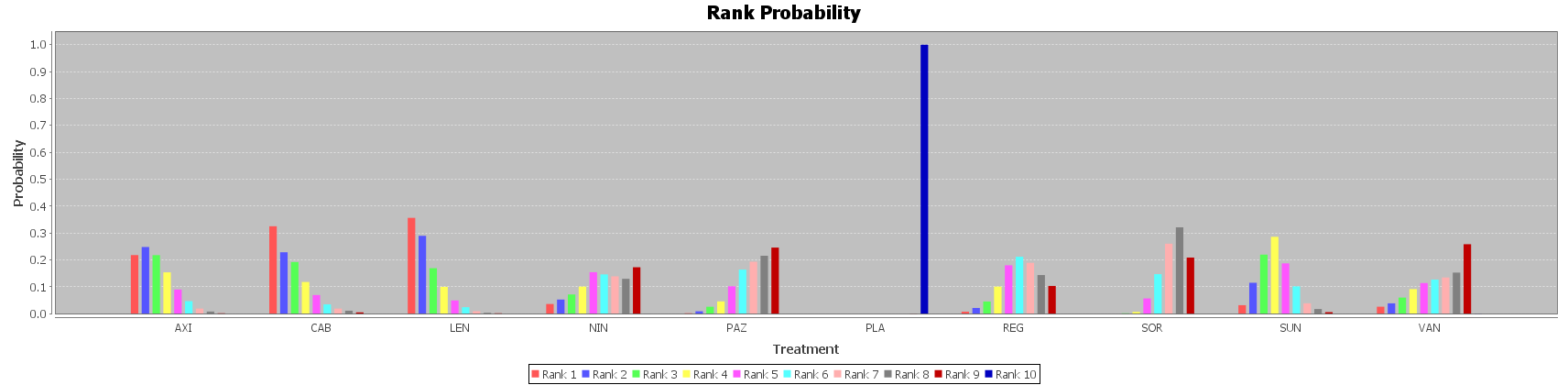

Supplement: Supplementary file 11 — Supplementary file11 (PNG 20 KB) [file 432_2021_3521_MOESM11_ESM.png]

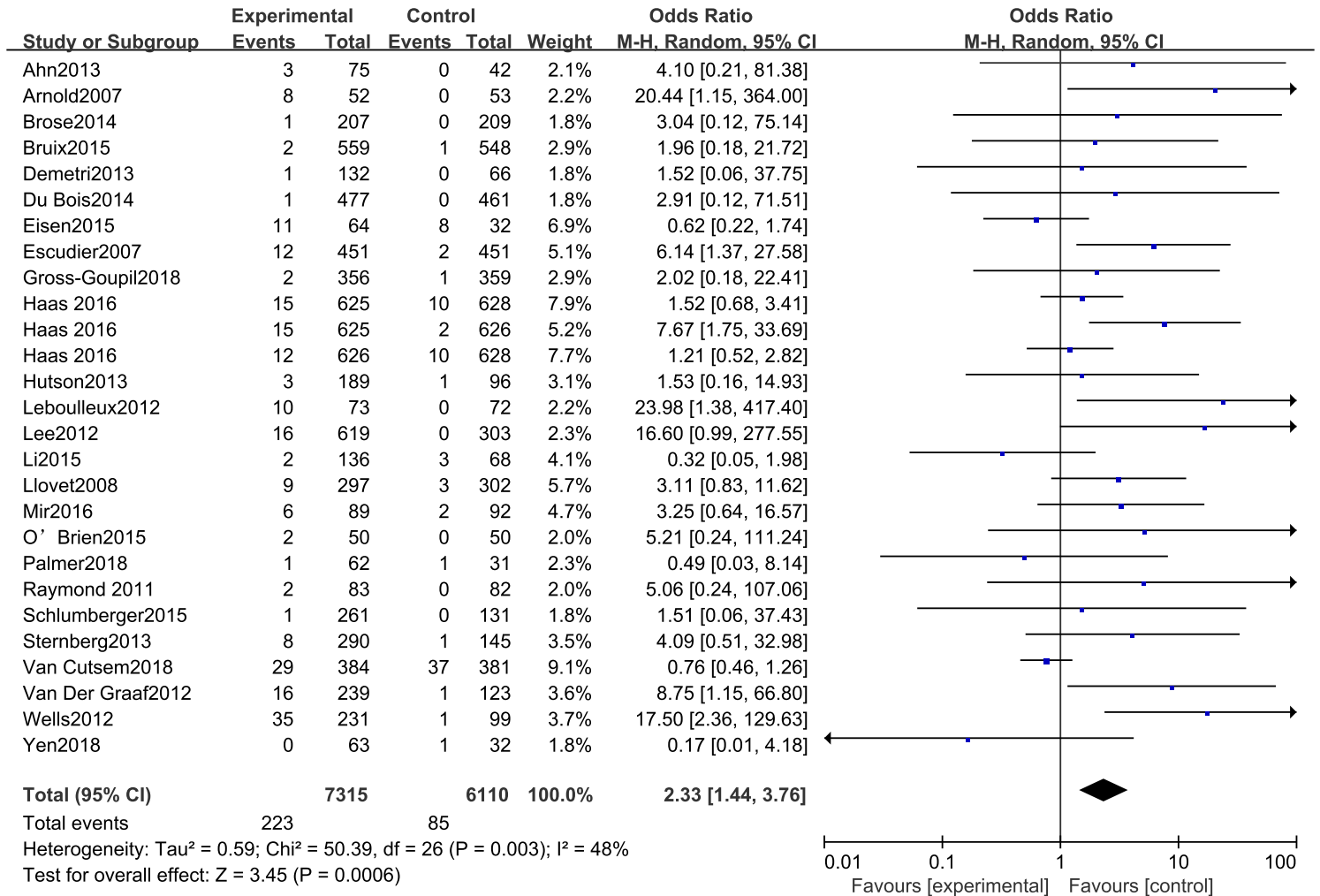

Supplement: Supplementary file 12 — Supplementary file12 (PDF 935 KB) [file 432_2021_3521_MOESM12_ESM.pdf]

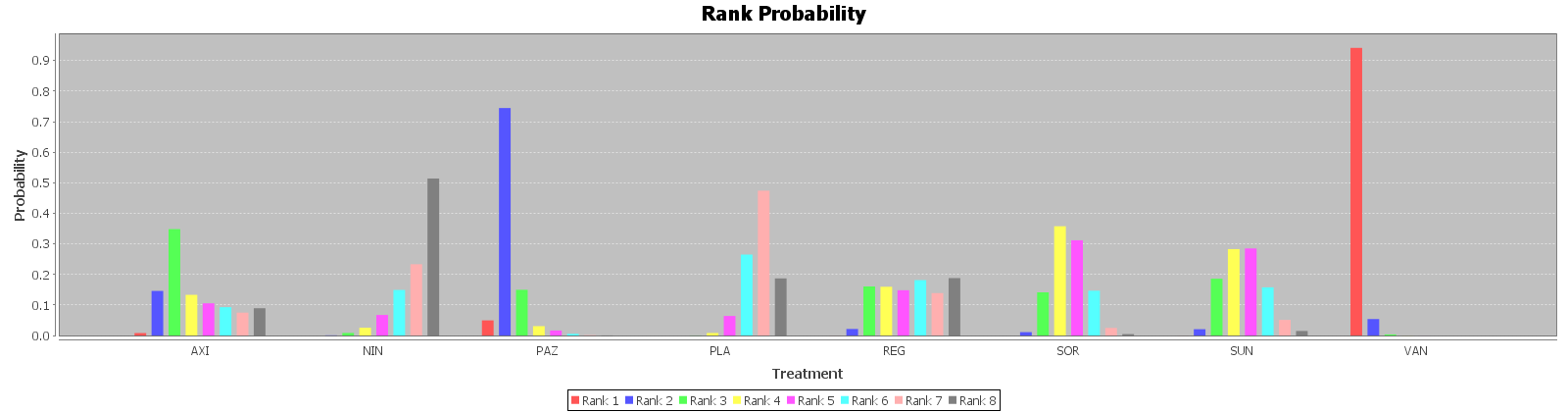

Supplement: Supplementary file 13 — Supplementary file13 (PNG 18 KB) [file 432_2021_3521_MOESM13_ESM.png]
